# Intervention Programmes for First-Episode Psychosis: A Scoping Review

**DOI:** 10.3390/nursrep15010016

**Published:** 2025-01-09

**Authors:** Marta Gouveia, Tânia Morgado, Tiago Costa, Francisco Sampaio, Amorim Rosa, Carlos Sequeira

**Affiliations:** 1 Local Health Unit of Viseu Dão-Lafões, 3504-509 Viseu, Portugal; 2 Abel Salazar Biomedical Sciences Institute, University of Porto, 4050-313 Porto, Portugal; 3 RISE-Health, Nursing School of Porto, 4200-450 Porto, Portugal; tmorgado@gmail.com (T.M.); tiagofilipeoliveiracosta@gmail.com (T.C.); franciscosampaio@esenf.pt (F.S.); carlossequeira@esenf.pt (C.S.); 4 Pediatric Hospital of the Local Health Unit of Coimbra, 3000-602 Coimbra, Portugal; 5 Health Sciences Research Unit—Nursing (UICISA: E), Nursing School of Coimbra, 3000-232 Coimbra, Portugal; amorim@esenfc.pt; 6 Nursing School of Coimbra, 3000-232 Coimbra, Portugal; 7 School of Health Sciences, Polytechnic of Leiria, Campus 2, Morro do Lena, Alto do Vieiro, Apartado 4137, 2411-901 Leiria, Portugal; 8 Local Health Unit of Gaia e Espinho, 4434-502 Vila Nova de Gaia, Portugal; 9 Red Cross Northern Health School, 3720-126 Oliveira de Azeméis, Portugal; 10 Nursing School of Porto, 4200-072 Porto, Portugal; 11 Research Unit, Nursing School of Porto, 4200-072 Porto, Portugal

**Keywords:** psychosis, early intervention, mental health, psychotic disorders, review

## Abstract

The aim of this scoping review was to map intervention programmes for first-episode psychosis by identifying their characteristics, participants, and specific contexts of implementation. It seems reasonable to suggest that early intervention may be beneficial in improving recovery outcomes and reducing the duration of untreated psychosis (DUP). Despite the expansion of these programmes, there are still some significant variations and barriers to access that need to be addressed. In line with the Joanna Briggs Institute (JBI) methodology and the Participants, Concept, and Context (PCC) framework, this review encompasses studies focusing on individuals grappling with early-stage psychosis and their caregivers across a range of settings, including hospital and community environments. The review identified 47 studies from 2002 to 2023, which revealed a great deal of diversity in programme characteristics and implementation contexts. This reflects a global perspective. The results showed that there is a great deal of variety in the characteristics of the programmes, with interventions ranging from single-component strategies, such as cognitive–behavioural therapy (CBT) and cognitive remediation therapy (CRT), to multicomponent programmes that integrate a number of different approaches, including psychosocial, pharmacological, and family-focused strategies. The objectives included attempts to improve cognitive functioning; enhance coping skills; reduce caregiver burden; and address symptoms such as anxiety, depression, and hallucinations. It is notable that there was considerable variation in the frequency, duration, and follow-up periods of the interventions, with some lasting just three sessions over one month and others spanning five years and 48 sessions. The majority of the programmes were delivered in community or outpatient settings, although there were also examples of hospital- and home-based interventions. These findings highlight the value of early interventions and provide a useful resource for adapting programmes to different social and cultural contexts. It would be beneficial for future research to explore how these interventions can be tailored to diverse settings.

## 1. Introduction

Psychosis is characterized by various signs and symptoms, including changes in thinking and perception [1]. Common features include delusions, hallucinations, mood disturbances, cognitive impairments, and behavioural changes [2,3,4]. The first psychotic episode generally occurs in late adolescence or early adulthood and is defined as the initial experience of such symptoms, lasting for at least one week and significantly impacting the individual’s functioning [3,4].

Early intervention in psychosis is widely recognised as essential for a favourable prognosis [5,6,7]. In recent decades, research in the field of early psychosis intervention and related therapeutic strategies has expanded [8,9], challenging the traditionally negative outlook associated with psychosis [10].

Three key contributions to the field are particularly significant. Firstly, Birchwood’s [11] studies highlight the critical intervention period, identified as occurring within two to five years after symptom onset, during which intervention is most effective and after which the effectiveness of intervention diminishes [1,2,11,12]. Secondly, McGorry’s [13] research on the staging of psychotic illness suggests that appropriate interventions can delay or even prevent progression to more advanced stages [9,13,14,15]. Lastly, the duration of untreated psychosis (DUP) has been recognised as a crucial factor influencing the course of the illness, with shorter DUPs linked to more positive outcomes [16]. This understanding has led to the identification of distinct stages within the disorder, facilitating the development of targeted interventions and a preventive approach [2,12,13,14] to halt progression to more advanced stages [17].

Given the nature of psychosis and developments in the field, early intervention is considered fundamental [14] and more effective than general care [1,5,18,19]. Early intervention requires intervention that is appropriate to the stage of the disease, promotes recovery, and delays or prevents deterioration of the person [20]. It aims to achieve outcomes not only at the clinical level (such as symptom reduction), but also at the personal level in terms of developing a productive and meaningful life [19,21,22]. In this context, the family will play an important role, not only because of the impact that a psychotic break can have on their dynamics [23], but also by actively supporting the individual in the recovery process [24,25] and helping to prevent relapse and social isolation. Their involvement is crucial in addressing the challenges posed by the illness [25].

Since its inception in 1992 in Melbourne, Australia [26], early intervention services have expanded significantly worldwide [1,8,27]. In 2005, the World Health Organization and the International Early Psychosis Association came together in the “Early Psychosis Declaration” to identify the essential components of early intervention services for psychosis, emphasising the need for a broad and eclectic approach [28] to promote recovery (symptomatic and functional) and empower the individual experiencing their first psychotic episode [9,28]. Early intervention must be accessible and comprehensive, involving caregivers. This requires a multidisciplinary approach that incorporates pharmacological and psychosocial interventions to reduce the severity and impact of the disease [1,2,3,22,23]. By implementing appropriate interventions, the aim is to minimise the risk of relapse, enhance the individual’s functioning, and promote recovery as swiftly as possible [12,29,30].

The number of programmes is growing worldwide [21], and the results are encouraging in terms of symptom reduction, overall functioning, quality of life, and relapse reduction [4,31] by reducing DUP, optimizing treatment response, reducing family burden, treating comorbidities such as substance abuse, and preventing disease progression [32]. Currently, there are guidelines with recommendations for the development of these programmes like Orygen [2], Health Service Executive (HSE) [4], and the National Association of State Mental Health Programme Directors (NASMHPD) [33]. Early intervention teams share common goals, including minimising the DUP, developing integrated treatments, and involving families [2,3,4,29,33,34]. However, there is significant variability in how these programmes are implemented [31]. Despite scientific evidence showing that early intervention is more effective than general care [1,18], its dissemination is limited, particularly in low- and middle-income countries [35,36]. In these regions, the development of early intervention programmes is often much slower [33,37] compared to high-income countries, where such programmes are widely established [38].

In this sense, the unequal development of mental health services gives rise to inequalities in access to care, with services often being geographically dispersed [35,36]. Additionally, barriers stemming from health systems or the services themselves reflect inconsistencies in their implementation [39]. This issue is particularly evident in low-income countries, where mental health services are frequently underfunded, and the DUP is often longer, leading to poorer recovery outcomes.

To address these issues, it is essential to create conditions that facilitate the development of services [35,36,37,40]. Improvements in accessibility, equity, and treatment outcomes can only be achieved through systematic implementation within national health systems [32]. Furthermore, ethical considerations must be integrated into the development of care pathways for psychosis, given that access is contingent upon socio-cultural contexts and the structure of health services [41].

Nevertheless, existing early intervention services show significant variation in their delivery models [31], leading to uncertainty about how best to adapt and implement them across different contexts. This variability underscores the need for comprehensive mapping to ensure that services are effectively tailored to the unique demands of diverse healthcare settings, calling for context-specific approaches [42]. The heterogeneity of these services, combined with the complexity of care pathways for first-episode psychosis, often results in a lack of standardised psychometric data, reflecting the diversity of intervention programmes and recovery trajectories [43]. To address these challenges and enhance care models, it is essential to develop fidelity scales that standardise implementation while incorporating quality indicators into their dissemination. This approach will ensure consistent application and maintain the effectiveness of these models across diverse settings [42].

A scoping review of early intervention programmes for psychosis is essential given the considerable variation and lack of standardisation in the way these programmes are described and structured. Although many interventions are documented, information is scattered across different sources, making a comprehensive and coherent understanding of current approaches difficult. This fragmentation hinders a full overview of key programme characteristics, including the type of intervention, facilitators, objectives, frequency of use, context of implementation, and evaluation methods. A scoping review will systematically map the range of existing programmes and provide a broad overview of their structure and implementation in different contexts. Consolidating this information into a single document will facilitate a clearer understanding of the diversity of approaches, enabling future discussion and further research into their adaptability and potential impact in different settings.

A preliminary search of the Cochrane Database of Systematic Reviews, PROSPERO, MEDLINE, and JBI Evidence Synthesis revealed that, although studies on this topic exist, no systematic review has addressed the specificities and scope of early intervention programmes for psychosis. It is therefore crucial to map the characteristics of these programmes to support their development and dissemination. In this context, mental health nurses, with their strong background in evidence-based interventions, play a key role within multidisciplinary teams, applying a holistic approach that takes into account the patient’s social and family context. By increasing their involvement, a more collaborative environment can be fostered, enhancing both the accessibility and comprehensiveness of mental health care. The objective of this scoping review is to map the features of intervention programmes for first-episode psychosis, including their characteristics, participants, and implementation contexts, whether in hospital or community settings.

## 2. Materials and Methods

This review was conducted according to the Joanna Briggs Institute (JBI) methodology for scoping reviews [44]. The Preferred Reporting Items for Systematic Reviews and Meta-Analyses extension for Scoping Reviews (PRISMA-ScR) checklist was used as a structuring matrix [45]. The review protocol was registered in the Open Science Framework on 26 February 2022 [46] and was conducted according to an a priori protocol published in 2023 [47]. OSF Registration Doi: 10.17605/OSF.IO/ZY9QM.

### 2.1. Review Questions

The objective of this study was to map the landscape of early intervention programmes for individuals experiencing a first episode of psychosis. The central review question was:

What early intervention programmes are implemented for service users and their families experiencing first-episode psychosis?

To address this, the following sub-questions were explored:

- What are the characteristics of these intervention programmes? (e.g., programme name, objectives, frequency, type of intervention, facilitators, evaluation methods, and implementation context)
- In what contexts are these programmes implemented?
- Who is the target audience for the intervention programmes (patients and/or family members)?

### 2.2. Inclusion Criteria

This review follows the methodology proposed by the JBI for scoping reviews, utilising the Participants, Concept, and Context (PCC) framework to ensure a comprehensive and structured approach to the collection and analysis of evidence. The key elements of the PCC relevant to this review are outlined below [44,45].

#### 2.2.1. Participants

This review included studies that included people with symptoms associated with the early stages of psychosis. Terms such as “first episode psychosis”, “recent onset psychosis”, “early onset psychosis”, and “early psychosis” may be used to describe participants. The study will not include individuals diagnosed with organic psychosis.

There are no restrictions on the age or gender of participants, with the only inclusion criterion being diagnosis. The study will also include caregivers, defined as first- or second-degree relatives who provide care and support to or live with people with first-episode psychosis. Participants may be either carers, people with first-episode psychosis, or both.

#### 2.2.2. Concept

This review considered studies that explored intervention programmes specifically designed to address first-episode psychosis and early-onset psychosis. These programmes provide a variety of interventions, including psychotherapeutic (e.g., cognitive–behavioural), psychosocial, vocational, and psychoeducational approaches, aimed at assisting both individuals experiencing their first episode of psychosis and their family caregivers.

It is important to note that interventions delivered in a general manner (e.g., “treatment as usual”) during consultations not specifically designated for first-episode psychosis in the early phase are excluded from this review.

#### 2.2.3. Context

This scoping review will consider studies from all countries and settings. This includes both hospital and community environments. There will be no exclusion criteria. We will include studies conducted in both inpatient and outpatient settings, whether psychiatric or non-psychiatric. Interventions delivered by trained healthcare professionals within a clinical intervention context, whether face-to-face, telephone-based, online, or home-based, will be considered.

#### 2.2.4. Types of Sources

Quantitative, qualitative, and multi-method/mixed-method studies were included in the scoping review. Quantitative studies comprise observational research with descriptive, exploratory, and analytical designs. All systematic reviews were included, independently of the types of methods of search used, as well as experimental designs like quasi-experimental studies, randomized controlled trials, and non-randomized controlled trials. Grey or unpublished literature, such as theses, dissertations, reports, government publications, organizational papers, and guidelines, was included. Sources of information had no geographical or cultural limitations and were consistent with the author’s proficiency in English, Portuguese, Spanish, and French. 

### 2.3. Search Strategy

The search strategy was designed to identify relevant studies and reviews, both published and unpublished. We began with an initial search of MEDLINE (PubMed) and CINAHL (EBSCO) to identify terms related to the topic. To develop a comprehensive search strategy for MEDLINE with full-text access via PubMed, we included text words from the titles and abstracts and their index terms (see Appendix A). This search strategy was adapted for each information source to include all identified keywords and index terms according to the inclusion criteria. In addition, we examined the reference lists of the articles included in the review to identify additional relevant articles.

### 2.4. Source of Evidence Selection

The search encompassed a variety of databases, including the Web of Science Core Collection (ISI Web of Knowledge), MEDLINE with Full Text, CINAHL Complete, PsycINFO (accessible through EBSCOhost), Scopus, the Cochrane Library, and JBI Evidence Synthesis. Additionally, efforts to identify unpublished studies involved searching OpenGrey, a European repository, as well as MedNar.

### 2.5. Study Selection

The search results were imported into EndNote vX9 (Clarivate Analytics, Philadelphia, PA, USA), where duplicates were removed. Two independent reviewers assessed the titles and abstracts to ensure they aligned with the inclusion criteria. Articles were selected based on the relevance of their titles and abstracts, including those that lacked an abstract. The reviewers thoroughly analysed any articles that met the inclusion criteria or raised uncertainties.

After this initial assessment, the full texts of the selected citations that complied with the inclusion criteria were reviewed by the two independent reviewers. Any disagreements were resolved through discussion, or, if necessary, a third reviewer was consulted. Full-text citations of eligible studies were uploaded into the JBI System for the Unified Management, Assessment, and Review of Information (JBI SUMARI), developed by the Joanna Briggs Institute in Adelaide, Australia.

Full-text articles that did not meet the inclusion criteria were documented and presented in a PRISMA-ScR flowchart diagram [45]. Authors of articles without access were contacted, and those articles were excluded if access could not be obtained. Due to the volume of articles, those without detailed information on programme characterisation, particularly frequency, were excluded. Articles were retained if they provided, at minimum, general information on the characterisation of the participants (including programme name, intervention objective, frequency (at least two out of four), type of intervention and evaluation). Although some articles did not always provide clear information on intervention facilitators or implementation context, they were still included, even if the information was incomplete.

### 2.6. Data Extraction

The data from the articles selected for the scoping review were extracted by two independent reviewers using a data extraction tool as outlined in the review protocol [47]. The extracted information included comprehensive specifications regarding the intervention programmes examined. In the event of any discrepancies between the reviewers, these were resolved through discussion, and if necessary, a third reviewer was consulted. Furthermore, the authors of the articles were contacted to obtain any missing information, ensuring that additional data were acquired as required.

### 2.7. Data Analysis and Presentation

The text presents the data through visuals, narrative, and tables. It outlines general study information, participant characterisation, programme characterisation, and the implementation context.

General study information includes the author, year of publication, country of origin, type of study, and study objectives. Participant characterisation covers the diagnosis, age of participants, and target group. Programme characterisation encompasses the programme name, intervention objectives, frequency (including the number, duration, and periodicity of sessions, as well as the follow-up period), intervention type (strategy and content), facilitators, and evaluation methods.

The data also address the implementation context, specifying the geographical area, if mentioned, and describing the setting—whether residential, community-based, or outpatient. Where possible, this section also includes the number of participants, indicating whether the intervention was delivered in a group or individual setting.

### 2.8. Study Inclusion

**Figure 1:**
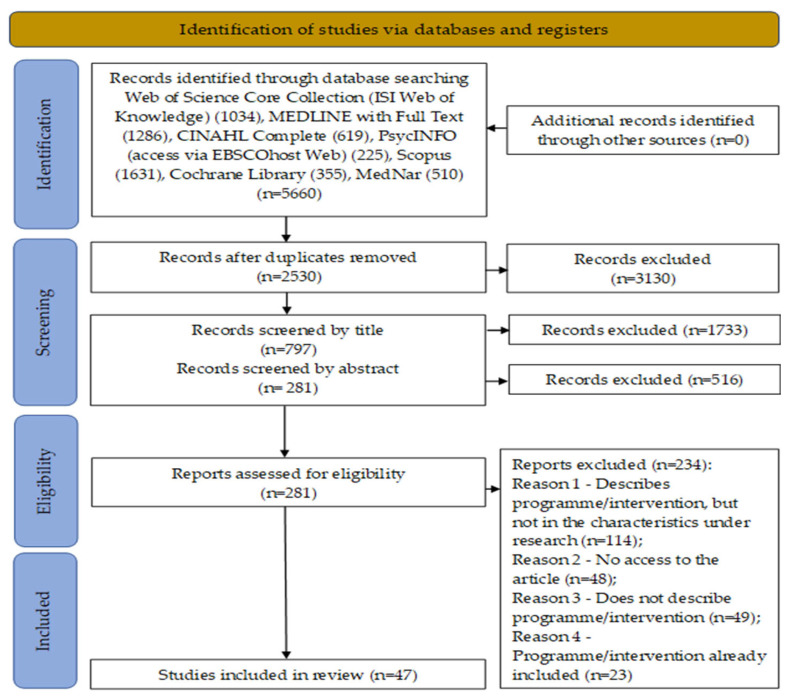
PRISMA flowchart of the screening and assessment process.

The process of searching and selecting evidence was followed as planned, and the results were synthesized into a PRISMA-ScR (Preferred Reporting Items for Systematic Reviews and Meta-Analyses extension for Scoping Reviews) flow chart, which can be viewed in Figure 1 [48].

## 3. Results Characteristics of Included Studies

### 3.1. General Study Information

This section summarises the general characteristics of the reviewed studies, including the author, publication year, origin, study type, and objectives (see Appendix B). The inclusion of 47 studies reflected a global perspective on early psychosis interventions. Publications spanned from 2002 to 2023. Contributions came from Australia (14.89%) [49,50,51,52,53,54,55], Canada (10.64%) [56,57,58,59,60], and several other countries: China (4.25%) [61,62], Croatia (2.13%) [63], Denmark (4.25%) [64,65], France (2.13%) [66], Germany (2.13%) [67], Iceland (2.13%) [68], India (2.13%) [69], Ireland (6.38%) [70,71,72], Italy (6.38%) [73,74,75], the Netherlands and Belgium (2.13%) [76], Norway (2.13%) [77], Singapore (2.13%) [78], Spain (10.64%) [19,79,80,81,82], Switzerland (2.13%) [83], United Kingdom (17.02%) [84,85,86,87,88,89,90,91], and the United States (6.38%) [31,92,93]. Studies originated from North America (n = 8), Europe (n = 27), Asia (n = 5), and Oceania (n = 7), with no representation from Africa or South America. Various research designs were used. Randomized controlled trials (RCTs) were predominant, including thirty-two studies [49,50,51,53,54,55,56,57,58,61,64,65,68,77,79,80,81,84,85,87,88,93]. Variations included Pragmatic Cluster RCT [73], Multicentre RCT [19], and Pilot RCTs [60,67,70]. Other designs were descriptive studies [52,59,63,66,74,75,78,82,83,92], cross-sectional studies [90], literature reviews [31], prospective controlled trials [76,86], and comparative studies [71]. Experimental designs featured quasi-experimental [69] and waiting list-controlled studies [62]. The objectives varied widely. Some studies explored cognitive interventions, compared therapeutic conditions (e.g., cognitive–behavioural therapy (CBT) combined with treatment as usual (TAU) vs. TAU alone), and evaluated multi-component psychosocial interventions by identifying barriers to feasibility and predictors of treatment effectiveness in first-episode psychosis (FEP) [19,73]. Others piloted programmes like group-based Integrated Cognitive Remediation (ICR) [68] or assessed novel interventions such as the Actissist mobile app [84]. The efficacy of peer-led family support versus traditional family psychoeducation and TAU was also examined [61]. The impact on cognitive functioning, social recovery, depressive symptoms, self-esteem, and quality of life was assessed [77,87,88]. Studies also investigated combining pharmacological and psychological interventions [85,86] and explored cost-effectiveness and satisfaction [50,67]. Additional research evaluated novel psychosocial interventions combining cognitive remediation therapy (CRT) and CBT [70], and RCTs assessed CBT for cannabis cessation [79] and cognitive therapy for suicidal patients [54], while the benefits of cognitive remediation on secondary negative symptoms and social functioning were also explored [93].

### 3.2. Participant Characterisation

The diagnoses considered included FEP (equivalent to recent-onset psychosis, early psychosis, early-onset psychosis, and first episode of schizophrenia spectrum disorder), which corresponded to studies that primarily focused on FEP patients (87.50% of the articles). The remaining 12.50% also referred to FEP patients, but the target group exclusively comprised the caregivers [50,52,55,62,69,90].

The age of the patients ranged from 12 [74] to 65 years [76,77,89], with different intervals depending on the article. The most common age range was 18–35 years, with 10.42% of articles, followed by 18–45 years, with 8.33%. Twenty-four articles dealt with the age group below 18 years [31,49,51,53,54,56,59,62,66,67,70,74,75,76,78,79,81,84,85,86,87,88]. Some articles did not specify the age (e.g., [63,64,71]), and three articles included the age group of over 65 years [76,77,89].

Of the selected articles, twenty-three included interventions only for patients, eighteen included interventions for both carers and patients, and six included interventions only for carers [50,52,55,62,69,90] (see Appendix C).

### 3.3. Programme Characterization

The included studies presented different interventions in terms of their objectives, frequency, intervention types (strategies and content), facilitators, and evaluations (see Appendix D).

#### 3.3.1. Programme Name/Intervention Objective

Given the high number of articles, an effort was made to group them into single-component interventions (e.g., CBT, computerised interventions, cognitive remediation, psychoeducation) or those that integrated multiple components (e.g., CBT + CM + psychoeducation). This categorisation was adopted for two primary reasons: to facilitate the organisation and analysis of the articles and to highlight the programmes’ characteristics. The classification provides a clear framework for presenting programmes with varying levels of complexity, which allows us to underscore the depth of focused interventions and the breadth of integrated programmes without overwhelming the reader with unstructured details. The included studies encompassed different psychosocial interventions, reflecting a wide array of cognitive, behavioural, and social approaches tailored to varying objectives and characteristics. It is acknowledged that some single-component interventions may have been part of broader programmes; however, they were analysed independently when the study’s primary focus was on a single component, as specified in the original articles.

Objectives: The core aim of each programme is to address a specific aspect of psychosis, such as improving cognitive function; enhancing coping skills; alleviating caregiver burden; or reducing symptoms like anxiety, depression, or hallucinations. These objectives are the intended outcomes of the intervention, guiding its design and scope.

Appendix E provides a brief overview of the different programmes, including their names (if assigned), intervention objectives, and whether they are single- or multi-component. Note that there may be selected articles in which only a single isolated intervention is analysed, which might be part of a broader programme (e.g., [54]).

##### Single-Component

Single-component interventions primarily focus on cognitive and cognitive–behavioural therapies. CBT is a prominent feature of numerous studies [51,57,67,71,76,77,79], addressing a range of objectives, including the reduction in both positive and negative symptoms, enhancement of overall functioning, support for cannabis cessation [79], and management of social anxiety [57].

In the realm of cognitive interventions, CRT is a central focus, with several studies indicating its efficacy in improving cognitive function and supporting functional recovery [78,86,87,93]. The objective of CRT is to enhance cognitive abilities and provide strategies to manage cognitive deficits. In addition to CRT, compensatory cognitive training (CCT) is a key element of this approach, intending to develop new cognitive habits to adapt to impairments [60].

Several studies have explored the potential of recovery-focused interventions, including bibliotherapy and problem-solving techniques. The use of bibliotherapy has been demonstrated to provide support to caregivers and alleviate psychological distress [50,55], and psychoeducation has been shown to assist patients and families in the management of early-onset psychosis [81]. Furthermore, the Method of Levels therapy [89] seeks to resolve goal conflicts and enhance self-management, while the Cognitive Recovery Intervention [88] is designed to reduce trauma symptoms and boost self-esteem or detect and monitor suicide-risk patients [54].

Other interventions include mindfulness-based social cognition training [80], which promotes a non-judgemental approach to interpersonal relationships, and psychoeducation [52,62,90], which enhances carers’ understanding of psychosis. A noteworthy innovation is a computerised approach to managing psychosis in real time [84].

##### Multicomponent

Multicomponent interventions integrate multiple therapeutic strategies to offer a comprehensive approach to early psychosis management. Programmes such as Cognitive Adaptation Training (CAT) and Action-Based Cognitive Remediation (ABCR) combine home-based supports with computerised cognitive exercises to address cognitive and motivational issues [56]. The Cognitive Remediation and Social Recovery in Early Psychosis (CReSt-R) programme merges CRT with social recovery therapy to improve both cognitive and social functioning [70].

Integrated approaches are significant in early psychosis management. The NEUROCOM programme combines cognitive remediation with OPUS treatment, which includes social skills training, patient psychoeducation, and family interventions [64]. Similarly, the cognitively oriented psychotherapy for early psychosis—COPE—programme integrates cognitive/behavioural therapy with psychoeducation and case management to facilitate patient adjustment and prevent secondary morbidity [49]. The NAVIGATE programme offers a comprehensive package including family education, individual resiliency training, and supported employment and education [92]. The Parma-Early Psychosis (Pr-EP) programme also includes a multi-component psychosocial intervention [74].

Family-focused interventions are crucial, with programmes like the Family-Led Mutual Support Group (FMSG) combining family psychoeducation with support groups to enhance family functioning and reduce rehospitalisation rates [61]. The Integrated Treatment Programme includes assertive community treatment (ACT), social skills training, and multifamily groups to address psychotic and disorganised symptoms [65]. The comprehensive therapeutic programme (CTP) utilises a range of therapies, including psychodynamic group psychotherapy, cognitive–behavioural workshops, and occupational therapy, to achieve remission and recovery [63].

Extensive programmes also play a vital role. The DETECT initiative combines CBT with occupational therapy and a Carer Education Programme to address early-phase psychosis and improve care [72]. The PEPP programme integrates cognitive skills training, family support, and individual therapeutic interventions to prevent relapse and support recovery [59]. The POTENTIAL programme employs a multidisciplinary approach, including individual and group therapies, to prevent chronic mental illness [31].

The combination of CBT and psychoeducation targets clinical improvement through normalising information, problem-solving, and relapse prevention [85] or enhancing functioning, treatment adherence, and illness awareness [19]. The Integrated Need-Adapted Treatment programme focuses on individual psychotherapy, group therapy, and improving treatment adherence [82]. Combining CR with CBT aims to enhance cognitive skills and facilitate effective symptom management [91]. The psychoeducational/psychosocial management approach targets social support and reduces family burden [69].

The EPPIC programme employs a multi-modal therapeutic approach, including relapse prevention and family-based CBT to prevent relapse after a first episode of psychosis [53]. The NEAR programme (Neurocognitive Educational Approach to Remediation) incorporates cognitive remediation, CCT, and social cognition and interaction training (SCIT) [68]. The Re-Arms programme combines CBT, case management (CM), and psychoeducation to improve overall treatment outcomes [75]. Additionally, the CBT plus psychoeducation approach [66] targets a reduction in psychotic symptoms and aims for a greater improvement in overall functioning, while ACT combined with case management [83] enhances continuity of care and reduces inpatient admissions.

Moreover, group treatments such as CBT for psychosis (CBTp) and symptom management (SM) programmes aim to enhance multiple protective factors, including skills, social competencies, family and social support, adaptive strategies, self-esteem, stress management, and medication compliance [58]. The AVEC component empowers families to support each other and provides information on various aspects of psychosis, contributing to the holistic approach to treatment and support.

The combination of CBT, case management, and family intervention for psychosis (FIP) enhances functioning, treatment adherence, and understanding of the condition, with a more substantial reduction in depressive, negative, and general psychotic symptoms following treatment [73].

#### 3.3.2. Frequency

The reviewed articles present a wide range of intervention programmes for psychosis, with considerable variation in the number of sessions (NS), treatment duration (TD), session frequency (FS), and follow-up (FU). The number of sessions varies significantly, with some programmes offering as few as 3 sessions [90], while others have up to 48 sessions [93]. Treatment durations also vary greatly, ranging from one month [52] to five years [82]. The frequency of sessions is similarly varied, ranging from daily [84] to fortnightly [19] or even monthly [80]. Follow-up periods are also inconsistent, with some studies having no follow-up (e.g., [52,68,78]), while others extend for up to 24 months or more after treatment [67].

Due to the wide heterogeneity of values for NS, TD, FS, and FU, the characteristics were described with a presentation of their amplitudes (minimum and maximum values) to illustrate the variation in the data, when possible. It is important to note that several articles did not report all relevant details, such as the duration of the sessions (e.g., [53,76]) or the exact follow-up periods (e.g., [66]), highlighting the need for more consistent reporting in the literature. In addition, a subset of articles (e.g., [63,89,92]) describes comprehensive interventions that are highly adaptable, with the number and frequency of sessions tailored to individual needs. These flexible programmes are common in cognitive–behavioural (CBT) and metacognitive training settings, emphasising personalised care to address both clinical and psychosocial concerns.

Given the large number of articles and the variation in the reported data, this summary provides an overview at a global level. More detailed information can be found in Appendix D.

#### 3.3.3. Intervention Type—Strategy/Content

Intervention programmes for psychosis include a wide range of strategies to support people at different stages of their illness.

Strategies and Contents: These refer to the specific therapeutic approaches and tools used to achieve the intervention’s objectives. For instance, CBTp focuses on the management of psychotic symptoms through structured phases, including goal setting and relapse prevention (e.g., [67]). Alongside CBTp, family interventions provide psychoeducation and support to families, aiming to enhance their ability to effectively manage psychosis-related challenges and foster resilience in the home environment (e.g., [61,69,92]). Comprehensive models, such as the NAVIGATE programme, combine various elements, including family education programmes (FEP), individual resilience training (IRT), supported employment and education (SEE), and individual medication management, to create tailored treatment plans [92]. These strategies promote recovery by integrating personal, social, and vocational support to address the multifaceted needs of patients. Cognitive remediation approaches like SCIT and CCT are aimed at improving cognitive functioning and social skills, directly addressing the cognitive deficits often associated with psychosis and complementing other therapeutic strategies such as CBTp or family support interventions [68].

The combination of psychoeducation and caregiver support programmes provides essential information and practical strategies to caregivers, which are critical for managing the long-term effects of psychosis on both the individual and their loved ones [69,72]. These strategies aim to reduce caregiver burden and improve overall care. Given the number of articles, this summary provides a general overview of strategies. More detailed information can be found in Appendix D.

#### 3.3.4. Intervention Facilitators

Interventions for psychosis involve a wide range of professionals, each with specific roles and different qualifications. Clinical psychologists are often associated with CBT and receive regular supervision to ensure the effectiveness of interventions (e.g., [63,67,77]). Psychiatrists play a crucial role in medication management and therapeutic support, collaborating with the team (e.g., [66,72,74]). Mental health nurses are particularly prominent in community and family therapy interventions. They provide psychoeducation, facilitate support groups, and manage cases, although they usually do not deliver CBT directly (e.g., [52,69,75,89]). Occupational therapists and social workers also play an important role in cognitive rehabilitation and psychosocial support (e.g., [61,64,80]).

Articles focusing on single-component interventions focus on specific techniques requiring specific training for each approach. In contrast, multi-component interventions are a combination of different methods and reflect a broader integration of professionals. Many articles do not fully describe the training or role of facilitators, but when they are mentioned, they are health professionals, often with specific experiences of psychosis. Appendix D provides more information about these practices and facilitator training.

#### 3.3.5. Evaluation

The selected articles yielded a considerable number of scales for the assessment of variables about mental health. Given the considerable diversity and quantity of instruments encountered, grouping these scales into categories was deemed appropriate, thus facilitating data analysis and interpretation. The categories were formed based on the key areas of assessment, which included: psychiatric symptom evaluation, functional assessment, cognitive assessment, well-being and quality of life assessment, and family and social support assessment (see Appendix F).

The category most frequently utilised was that of psychiatric symptom evaluation. The Positive and Negative Syndrome Scale (PANSS) and the Brief Psychiatric Rating Scale (BPRS) were identified as the most recurrent scales within this category. These instruments were used extensively for the assessment of symptom presence and severity. In the category of functional evaluation, instruments such as the Global Assessment of Functioning (GAF) and the Social and Occupational Functioning Assessment Scale (SOFAS) were employed with considerable frequency. These tools assess the impact of mental disorders on people’s daily lives, providing an integrated view of social and occupational functioning.

In the context of cognitive assessment, the Wechsler Adult Intelligence Scale (WAIS) and the Cambridge Neuropsychological Test Automated Battery (CANTAB) were frequently referenced. These scales are used to assess patients’ cognitive abilities. This is an area of growing interest in psychiatry. Moreover, the domain of well-being and quality of life was a significant area of emphasis, with the EuroQol 5-Dimensions 5-Levels (EQ-5D-5L) and the World Health Organization Quality of Life (WHOQOL-Bref) being the most frequently referenced instruments. These instruments are employed to ascertain the patients’ perceptions of their quality of life. In several articles, the use of any scales was not specified. Moreover, several studies opted to employ a combination of scales to gain a more comprehensive understanding of the subject matter, thereby highlighting the intricate nature of the topic under investigation (See Appendix D).

### 3.4. Implementation Context

In examining the implementation contexts of various interventions, many studies were conducted in urban settings [50,51,61,68,71,72,76,85,88,93]. Research in semi-rural and rural areas comprised five studies [54,57,73,74,84]. Additionally, a substantial number of studies did not specify their implementation contexts [19,31,49,52,53,55,56,58,59,60,62,63,64,65,66,69,70,75,77,78,79,80,81,82,83,85,86,87,89,90,91,92].

#### 3.4.1. Setting

The analysis identified three primary settings for interventions. Inpatient settings involve interventions conducted within hospitals or residential facilities [59,69,83,87]. Home-based settings deliver interventions within patients’ homes [49,50,51,56,76,84,91]. Community-based and outpatient settings utilise local resources and encompass interventions conducted in clinics, community centres, or other non-residential environments [19,31,52,53,54,55,57,58,59,60,61,62,63,64,65,66,67,68,71,72,74,75,77,78,79,80,81,82,83,85,86,87,88,89,90,92,93]. Notably, only one review addressed interventions in both inpatient and outpatient settings [83].

#### 3.4.2. Individual/Group Intervention

Additionally, interventions varied in their session formats. Some were exclusively individual [86,89], while others included both individual and group sessions [50,59,81]. Group sizes ranged from small (4 participants) to larger groups (up to 15 participants) [78,80,86]. Community-based and outpatient interventions often featured group formats but did not always specify the number of participants [52,90]. Overall, while individual interventions were predominant, several studies incorporated both individual and group sessions, tailored to the needs of participants and the specific setting.

Individual interventions involve therapeutic approaches delivered on a one-on-one basis between the therapist and the patient [19,49,51,53,54,55,56,58,59,62,63,66,67,70,75,76,77,78,79,80,81,83,84,85,86,87,88,89,93]. Group interventions describe therapeutic approaches delivered to multiple participants simultaneously in a group setting [52,58,60,61,62,63,65,69,71,72,80,86,87,90]. Mixed individual and group interventions combine elements of both individual and group therapy [31,50,51,53,58,59,61,62,63,64,65,68,73,74,81,83,88]. Several articles do not specify whether the intervention is individual or group-based [19,31,52,53,54,55,56,57,59,63,64,66,67,74,75,77,78,79,80,81,82,83,88,89,90,91,92]. For detailed information, see Appendix D).

## 4. Discussion

The scoping review provides a detailed overview of early intervention programmes for first-episode psychosis, revealing considerable diversity in their characteristics, participants, and delivery contexts. To the authors’ knowledge, this is the first review to map programmes along these lines. Of the 47 articles included, there was variation in terms of the structure and type of study. On the other hand, their focus—intervention programmes—also varied in terms of objectives, types of intervention, and implementation strategies. They therefore reflect the inherent complexity of psychosis [94] and the diverse needs of individuals and their families, as highlighted in studies on the staging models of psychosis [13] and on the needs of patients and their families [32].

The findings confirm the importance of a comprehensive and tailored approach to psychosis treatment, aligning with previous research advocating for interventions designed to meet specific manifestations of the illness [13,95]. There is widespread agreement in the literature on the superiority of intensive, team-based interventions for FEP compared to TAU [18,94]. Throughout our review, we can see that, of the 23 articles identified as single-component, 16 are integrated into broader therapeutic programmes where, in addition to the intervention under study, other specific psychotherapeutic or psychosocial interventions for FEP may already be available [50,51,54,55,57,60,62,78,80,86,88,89,90,93]. This finding is supported by recent research highlighting the effectiveness of integrated approaches in treating complex psychopathology. The analysis by Williams et al. reinforces the effectiveness of integrated techniques, showing that models that combine a comprehensive package of treatments are more effective in optimising outcomes and meeting the specific needs of patients in the long term, suggesting that, although some interventions may appear isolated, they are often part of more complex and multifaceted therapeutic contexts [94]. Examples include PSBI (problem-solving therapy) [50,55], which is part of the specialist FEP centres Orygen Youth Health (OYH) and the Recovery and Prevention of Psychosis Service (RAPPS), or even CRT for psychosis, which is part of the NHS early intervention services in the UK [86]; CRT is part of the Early Psychosis Intervention Programme (EPIP) in Singapore [78].

Among the selected articles, many included interventions aimed at carers. These family interventions were frequently associated with reported benefits, such as a reduction in caregiver burden and positive effects over time [96]. Additionally, the studies indicate that these interventions are linked to potential improvements in outcomes such as reduced relapse rates, shorter hospital stays, and psychotic symptoms, as well as improved functionality in patients with first-episode psychosis up to 24 months following the intervention [97]. It has therefore been suggested that these interventions should be incorporated into mental health services, as they offer benefits such as reducing the burden on carers and improving their emotional well-being, as well as helping them to cope with challenges such as the uncertainty and stigma associated with caring [98]. Despite the emphasis in the literature on the need for a holistic and integrated approach [98], in our analysis, 24 articles focused on carers, which is consistent with Claxton et al. [99], who suggest that carers’ needs and the emotional impact of caring remain areas that are often neglected.

In terms of the age groups covered, they vary, but are mainly focused on adolescents and young adults, with varying ranges, which is in line with McGorry et al. and Fusar-Poli et al., who inform us about a youth-oriented intervention [32,100]. In our case, the age range of 14–35 covers the largest number of studies. Some articles cover a wider population, including ages as young as twelve or as old as sixty-five. This reflects the importance of early intervention, particularly during the transition from adolescence to adulthood, and is in line with Addington, who tells us that early intervention for psychosis should take place from early adolescence to adulthood, with a gradual decline up to the age of sixty [101]. Others do not specify an age, suggesting a more comprehensive or generalist approach to intervention. Overall, the diversity of age groups highlights the need for intervention strategies adapted to different phases of life [102]. The health benefits also exist for later implementation, returning to the example of the UK, where guidelines have extended the age of eligibility to sixty-five [103].

In terms of interventions, CBT, CRT, and psychoeducational interventions stand out. CBT was widely used, appearing alone in several articles [51,57,67,71,76,77,79] as well as in combination with other modalities such as cognitive remediation, psychoeducation, and case management [19,49,53,58,66,72,73,74,75,85,91]. Combinations including elements of CCT and mindfulness-based therapies have also been identified [68,80]. This diversity reflects growing evidence in the literature suggesting that integrated approaches are frequently associated with better management of psychotic symptoms and promotion of functional recovery, as they address both cognitive and behavioural aspects [98,104].

These data are consistent with Gergov et al., who report that there are benefits to offering cognitive, behavioural, or CBT and CRT psychotherapeutic interventions to patients, to carers, or in group settings, especially when psychoeducational elements are included [105]. In addition, multi-component programmes such as NAVIGATE [92] and POTENTIAL [31] reinforce the importance of comprehensive approaches that integrate multiple therapeutic techniques and synergistically address the diverse needs of patients.

According to Breitborde et al., optimising interventions in the first psychotic episode also involves a synergistic combination of interventions [106]. In this sense, psychoeducation is a component of evidence-based intervention, as is case management with a comprehensive approach to the patient’s needs [101,107]. Interventions such as OPUS [65], which combines case management with social skills training and multi-family groups, are examples of this integrated approach. They aim for both clinical stabilisation and social reintegration. On the other hand, CBT is also effective in reducing positive symptoms, while family interventions are effective in preventing relapse [108]. Williams et al. suggest that psychological interventions and case management, together with pharmacotherapy, are the central components of services for early psychosis to achieve sustained clinical benefit [94].

These findings suggest that the diversity of interventions reflects the complexity of the treatment of psychosis. To improve outcomes in a complex and heterogeneous syndrome such as psychosis, it is necessary to employ complex intervention models globally [32]. This comprehensive focus is essential given that early intervention services for psychosis are effective in the broader context of mental health care, which is supported by various guidelines such as the Australian Clinical Guidelines for Early Psychosis [2]. At the educational level, interventions aim to improve knowledge about the disease and its treatment, both for patients and their carers, to facilitate treatment adherence and promote a supportive environment [19,31,52,54,58,59,62,63,72,73,74,81,82,83,85,89,90,91,92]. Finally, at the social level, the aim is to strengthen family and social relationships, improve communication and mutual support, and reduce the emotionality expressed in family interactions, which is crucial for successful recovery and maintenance of mental health [50,55,58,59,61,68,69,80,82,92].

In the analysis of the objectives of the interventions, there is an underlying multidimensional basis that aims to promote improvements at different levels. There is variability between programmes and types of interventions. This is consistent with findings in the literature that point to the existence of multiple approaches [18,107]. Our findings were consistent with these findings, with broad aims. Clinically, interventions aim to reduce positive and negative psychotic symptoms, improve cognitive function, and prevent relapse [19,31,49,51,53,56,57,58,59,60,61,63,64,65,66,67,68,70,71,72,73,74,75,76,77,78,79,82,83,84,85,86,87,89,91,92,93]. Functionally, the focus is on restoring social, occupational, and educational skills to enable meaningful reintegration into society [19,31,49,51,57,59,64,66,68,70,71,72,73,74,75,76,77,79,84,91,92]. Psychological aims include supporting emotional well-being, reducing psychological distress for both patients and carers, and promoting a more stable and less stressful family environment [31,49,50,54,55,56,57,59,67,69,72,73,74,75,76,77,80,81,82,83,88,89,90,92]. These goals highlight the importance of a holistic approach to recovery, which goes beyond symptom reduction and promotes an overall improvement in the quality of life and functioning of patients and their families. Fusar-Poli et al. refer to secondary prevention by highlighting the fact that services promote the reduction in SUD. In terms of interventions, they are based on improving the response to treatment, with improvements in well-being, functioning, and social skills, and reducing the burden on the family. They also promote the treatment of comorbid substance use and the prevention of disease progression [32]. However, although the objectives of the interventions imply health benefits, it is still unclear how they should be developed to enable their long-term maintenance [109].

When analysing the frequency and duration of interventions, the programmes varied considerably in terms of the number of sessions, length of treatment, duration of sessions, frequency of interventions, and follow-up periods, reflecting the complexity of treating psychotic illnesses and the need for individualised approaches. Our findings are consistent with Birchwood’s studies, which indicate the existence of a critical period of intervention, a period that can last up to 5 years, during which there is a possibility of achieving more fruitful results [11]. Thus, the existence of variability in frequency is also in line with Chan et al., who argue that interventions should be culturally adapted and tailored to the individual needs of patients, highlighting the importance of a personalised approach to treatment [110].

The variation in follow-up times highlights the importance of ongoing monitoring to assess the long-term effectiveness of interventions and ensure the sustainability of therapeutic gains. However, the variation in follow-up times reflects the lack of a standardised protocol and points to the need for future studies to explore the long-term effectiveness of interventions in order to better guide clinical practice [94].

Although early intervention in psychosis has positive short-term outcomes, there is still uncertainty about the maintenance of these benefits after five years of treatment [18,111]. These findings are in line with Hegelstad, who confirmed the benefits of early intervention but highlighted the need to identify strategies to maintain these benefits in the long term [112]. Favourable outcomes are not always maintained in the long term [111], especially when a transition to treatment as usual occurs [113].

Interventions are delivered by a wide range of professionals, including clinical psychologists, psychiatrists, mental health nurses, occupational therapists, and social workers. The training and supervision of these professionals vary widely, from specialist training in CBTp [49,58] to the implementation of intervention models such as NEAR [93,114] or CRT [86].

Most interventions include regular supervision, feedback sessions, and, in some cases, monitoring of adherence to the protocol (e.g., [85]), which has been associated with improved treatment efficacy [115]. It also suggests that the quality of the intervention may be directly related to the training and ongoing supervision provided to therapists. Furthermore, the presence of a multi-professional team, as in the ReARMS and OPUS programmes (e.g., [65,75]), highlights the importance of a collaborative and holistic approach to the treatment of psychosis. Nevertheless, there is considerable variation in the training of intervention facilitators. Some studies report intensive and specific training (e.g., [58]), while others mention minimal training or do not specify training criteria (e.g., [79]).

Regarding assessment, the results show that a wide variety of assessment instruments are used in early intervention programmes for psychosis. The diversity of scales, covering areas such as family functioning, quality of life, cognitive assessment, psychiatric symptoms, social and occupational functioning, anxiety, depression, self-esteem, and illness awareness, highlights the complexity of psychosis treatment [94]. The multiplicity of domains assessed highlights the need for a multidisciplinary and personalised approach to the quality of care provided [116]. However, the variability of the instruments used may also indicate a lack of standardisation, which can make it difficult to compare results between different studies and programmes [116]. Thus, these results suggest the importance of continuing to explore which tools offer greater sensitivity and specificity to assess the various dimensions of the psychotic experience, promoting a better understanding of the illness and interventions in its trajectory, ultimately improving treatment outcomes and guiding clinical practice. Regarding implementation contexts, it has been found that inpatient environments can be more disruptive in various psychosocial aspects, and, in the sense of recovery, it is advocated that inpatient stays occur as a last measure and for the shortest time necessary, with a smooth transition to care in the community (whether outpatient or community) (e.g., [59,83,87,117]), which is in line with the majority of the articles selected where the implementation context, although varied, takes place in outpatient settings (e.g., [58,64]), at home (e.g., [49,51]), and in the community (e.g., [66,74]).

Even so, according to Siebert and colleagues, specialised inpatient services can be an asset to effective global intervention in the event of the need for hospitalisation [118], and communication between inpatient services and subsequent outpatient follow-up is an indicator of quality [108].

Several limitations were identified in this review. Access to some full-text articles was not always possible, potentially excluding relevant studies. Only studies published in English, Portuguese, Spanish, and French were included, as these were the languages spoken by the authors. This ensured the quality of the review, but may have limited its scope. Additionally, while some studies lacked detailed information on programme characteristics, those with sufficient data to meet the review’s objectives were included. Finally, the decision to exclude interventions not specifically designed for early psychosis may have omitted broader approaches, though this was a necessary methodological choice.

## 5. Conclusions and Implications

This scoping review highlights the importance of early intervention in psychosis and maps the extensive existing research on appropriate interventions in this area. By identifying the characteristics of current programmes, we reveal a diversity of approaches and variability in implementation strategies. This mapping provides a valuable resource for adapting programmes to diverse political, social, and cultural contexts.

The implications of this mapping are considerable. It provides a solid basis for researchers and health professionals to explore interventions that can improve access to mental health care. Furthermore, this review contributes to the development of a specific early intervention programme designed specifically for mental health nurses. Such a programme would enhance their role in multidisciplinary teams and equip them with the tools to provide timely, patient-centred care. While the findings of this review emphasize the importance of multidisciplinary collaboration, they also highlight the unique contributions that mental health nurses can make in supporting holistic and inclusive care. To improve clinical practice and ensure high-quality mental health care, future research should focus on exploring the effectiveness of the mapped interventions and how they can be adapted to different contexts.

## Data Availability

Not applicable.

## Public Involvement Statement

There was no public involvement in any aspect of this research.

## Guidelines and Standards Statement

This manuscript was drafted against the Joanna Briggs Institute (JBI) methodology for scoping reviews as [44].

## Appendix A

**Table A1:** Draft of search strategy to Medline (PubMed) ^1^.

Search No.	Query	Records Retrieved
#1	Search: (“first episode psychosis “[Title/Abstract] OR “First-episode psychosis “[Title/Abstract] OR “first episode psychoses “[Title/Abstract] OR “First-episode psychoses “[Title/Abstract] OR “first episode of psychosis “[Title/Abstract] OR “First-episode of psychosis “[Title/Abstract] OR “first episode of psychoses “[Title/Abstract] OR “First-episode of psychoses “[Title/Abstract] OR “early onset psychosis “[Title/Abstract] OR “early onset psychoses “[Title/Abstract] OR “early psychosis “[Title/Abstract] OR “early psychoses “[Title/Abstract]) AND ((“Psychotherapy, Group “[Mesh] OR “Psychosocial Intervention “[Mesh] OR “Behavioral Symptoms “[Mesh] OR “Cognitive Behavioral Therapy “[Mesh] OR “Counseling “[Mesh]) OR (“early intervention “[Title/Abstract] OR “Group Psychotherapy “[Title/Abstract] OR “Group therapy “[Title/Abstract] OR “Cognitive behaviour “[Title/Abstract] OR “Cognitive behaviours “[Title/Abstract] OR “cognitive behavioral “[Title/Abstract] OR “Biopsychological interventions “[Title/Abstract] OR “Biopsychological intervention “[Title/Abstract] OR “Psychosocial Interventions “[Title/Abstract] OR “Psychosocial Intervention “[Title/Abstract] OR “Behaviour Therapy “[Title/Abstract] OR “Behaviours Therapy “[Title/Abstract] OR “behavioural therapy “[Title/Abstract] OR “Cognitive Restructuring “[Title/Abstract]))	1753

^1^ Search date: 28 December 2022

## Appendix B

**Table A2:** General study information.

Title/Author/Year of Publication/Country	Type of Study	Objectives
Kidd SA, et al. (2019, Canada) [56]	RCT	Investigate the use of cognitive interventions in the treatment of early psychosis, focusing on the comparative impacts of primarily compensatory versus restorative approaches.
Jackson H, et al. (2005, Australia) [49]	RCT	Compare COPE ^1^ plus standard EPPIC care versus standard EPPIC care alone (No-COPE).
Mueser, KT, et al. (2015, USA) [92]	Descriptive study	Outline the background, rationale, and specifics of the intervention created by the RAISE Early Treatment Program (NAVIGATE program), emphasizing the psychosocial components.
González-Ortega I, et al. (2021, Spain) [19]	Multicentre, single-blind, RCT	Compare the efficacy of CBT ^2^ combined with TAU ^2,3^ versus TAU alone for FEP ^3,4^. Assess the differences in BDNF ^4,5^ levels between the groups.
Ruggeri M, et al. (2012, Italy) [73]	Pragmatic cluster randomized controlled design	Assess the effectiveness of a multi-component psychosocial intervention compared to TAU at the nine-month mark. Identify challenges related to the feasibility of the intervention, and analyse how clinical, psychological, environmental, and service organisation factors influence treatment outcomes, adherence, and satisfaction in individuals with FEP.
McCann TV, et al. (2012, Australia) [50]	RCT	Evaluate whether self-directed problem-solving bibliotherapy, compared to TAU, enhances caregiving experiences, reduces distress and expressed emotion, and improves overall health.
Vidarsdottir OG, et al. (2019, Iceland) [68]	RCT	Conduct a pilot evaluation of a group-based ICR ^6^ programme incorporating SCIT ^5,7^, NEAR ^6,8^, and CCT ^7,9^.
Chien WT, et al. (2018, China) [61]	RCT	Assess the effectiveness of a peer-led FMSG ^8,10^ intervention and compare its outcomes with those of a family psychoeducation group programme and TAU alone.
Bucci S, et al. (2018, UK) [84]	RCT	Evaluate the safety, feasibility, and acceptability of the Actissist intervention. Provide preliminary evidence regarding its impact on clinical and functional outcomes.
Jackson H J., et al. (2007, Australia) [51]	RCT	Conduct an RCT comparing CBT with Befriending for patients experiencing the acute phase of their first episode of psychosis within a single treatment setting.
Morrison AP, et al. (2020, UK) [85]	RCT	Assess the feasibility of three approaches: antipsychotic monotherapy, monotherapy with psychological intervention, and a combination of antipsychotics with psychological intervention.
Müller H, et al. (2019, Germany) [67]	Multi-centre, prospective, single-blind randomized controlled pilot trial	Examine the acceptance, tolerability, feasibility, and safety of modified CBT and combined CBT with TAU, as compared to TAU alone.
Sönmez N, et al. (2020, Norway) [77]	RCT	Compare the effectiveness of CBT with TAU in reducing depressive symptoms and enhancing self-esteem, alleviating symptoms as measured by the PANSS ^10,11^, and improving overall functioning.
González-Ortega I, et al. (2016, Spain) [79]	RCT	Outline the study protocol design for an RCT aimed at evaluating the efficacy of a specific CBT programme for cannabis cessation compared to standard psychoeducation treatment.
Frawley E, et al. (2022, Ireland) [70]	Pilot Feasibility Study	Investigate the feasibility, acceptability, and effectiveness of a new psychosocial intervention that integrates CRT ^11,12^ and CBT, specifically targeting social recovery.
Wykes T, et al. (2018, UK) [86]	Multicentre, randomised, single-blinded, controlled trial	Determine the optimal method for delivering CRT by comparing intensive, group, and independent approaches. Assess effectiveness based on goal achievement, improvements in cognition, social functioning, self-esteem, symptom reduction, cost-efficiency, and satisfaction of service users and staff.
Østergaard Christensen T, et al. (2014, Denmark) [64]	RCT	Assess the impact of combining NEUROCOM with the OPUS early intervention service compared to the OPUS service alone. Analyse the effects on functional capacity, cognitive performance, symptomatology, and self-esteem.
Krarup Get T, et al. (2005, Denmark) [65]	RCT	Explore how the OPUS early intervention service influences symptoms of negativity, psychosis, and disorganisation.
Wykes T, et al. (2007, UK) [87]	RCT	Assess the efficacy of CRT ^12^ in reducing cognitive deficits compared to TAU and investigate the mediating and moderating effects of cognitive improvement.
Lepage M, et al. (2023, Canada) [57]	randomized controlled trial	Evaluate the efficacy of a group CBT intervention for SA ^13^, specifically designed for young individuals who have experienced a FEP.
Leclerc C, et al. (2012, Canada) [58]	RCT	Clarify the reasons behind the variability in results from CBTp and discuss why group therapy has yielded the most favourable outcomes. Present the findings from a combined approach involving CBT and psychoeducation for families. Additionally, compare the effects of three conditions: CBTp, SM ^12,14^, and TAU or control group.
Šago D, et al. (2018, Croatia) [63]	Descriptive study	Outline the establishment of the initial day hospital for early intervention and treatment at the Psychiatric Hospital “Sveti Ivan” in Zagreb, Croatia.
Sadath A, et al. (2016, India) [69]	Quasi-experimental nonequivalent comparison group design	Assess the impact of a group intervention on carers’ expressed emotion and social support and compare these effects with those of TAU.
So HW, et al. (2006, China) [62]	waiting list-controlled study	Assess the effectiveness of a brief psychoeducational intervention for carers. Measure changes in participants’ understanding of psychosis, caregiving burden, coping strategies, and expressed emotions.
Reininghaus U, et al. (2019, Netherlands and Belgium) [76]	multi-center randomised controlled trial study protocol	Investigate the efficacy of a novel ecological momentary intervention, Acceptance and Commitment Therapy in Daily Life (ACT-DL), for individuals with Ultra-High Risk (UHR) or experiencing a FEP.
Leuci E, et al. (2019, Italy) [74]	Desciptive	Outline the macroscopic organisation of the Pr-EP ^15^ initiative and analyse specific process indicators over the first five years since its inception.
Gaynor K, et al. (2011, Ireland) [71]	Comparative study	Examine whether there is an early critical period during which patients are particularly responsive to psychological treatment.
Turner N, et al. (2011, Ireland) [72]	Report	Advocate for increased dedication to early intervention strategies and support the establishment of such services in additional areas throughout Ireland.
Jackson C, et al. (2009, UK) [88]	RCT	Evaluate the efficacy of a specific form of CBT, known as cognitive recovery intervention (CRI), in alleviating trauma, depression, and low self-esteem.
Griffiths R, et al. (2019, UK) [89]	parallel group RCT design	Determine the feasibility and acceptability of MOL ^14,16^ and assess its potential for further evaluation in a clinical trial.
Mediavilla R, (2019, Spain) [80]	RCT	Compare the effectiveness of SocialMIND ^15,17^ on social functioning with that of a PMI ^16,18^.
Lecardeur L, (2018, France) [66]	Descriptive study	Outline the activities undertaken by a Mobile Intensive Care Unit in France.
Mullen A, et al. (2002, Australia) [52]	Descriptive study	Evaluate an MFG education programme developed for families.
Onwumere J, et al. (2017, UK) [90]	Cross-sectional design using pre-post measures.	Explore whether a short-term group intervention can improve negative perceptions of illness among carers of individuals experiencing their FEP.
Poletti M, et al. (2020, Italy) [75]	Descriptive	Describe the overall structure of ReARMS and analyse specific process indicators. Assess the feasibility and quality of its procedures, particularly for the subgroup of adolescents seeking help.
Gleeson J, et al. (2008, Australia) [53]	RCT	Determine whether relapse rates can be reduced through a multi-modal therapeutic intervention compared to TAU within a specialised FEP programme.
Power PJR, et al. (2003, Australia) [54]	RCT	Describe the development of LifeSPAN therapy, a cognitive treatment specifically designed for acutely suicidal patients, and its evaluation.
Calvo A, et al. (2014, Spain) [81]	RCT	Investigate the efficacy of a structured psychoeducational group intervention for adolescents experiencing early-onset psychosis and their families.
De Maio M, et al. (2014, USA) [31]	Literature review+ descriptive	Provide a detailed description of the POTENTIAL Early Psychosis Programme, including its model and rationale, and highlight the unique aspects of the programme.
McCann TV, (2015, Australia) [55]	RCT	Investigate whether self-directed problem-solving bibliotherapy completed by first-time carers of young individuals with a first episode of psychosis enhances their social problem-solving skills compared to carers who only received TAU.
Baumann PS, et al. (2013, Switzerland) [83]	Descriptive study	Detail the implementation of a specialised programme designed to enhance engagement and the quality of treatment for early psychosis patients in the Lausanne area of Switzerland.
Malla AK, et al. (2002, Canada) [59]	Descriptive study	Outline a holistic approach to managing FEP and report on the clinical outcomes after one year for an epidemiological cohort of patients with FEP who were treated within a specialised programme tailored to their specific needs.
Domínguez MT, et al. (2011, Spain) [82]	Descriptive	Present and outline the integrated, needs-based treatment approach being developed within the early psychosis programme at a specialised centre in Barcelona, Spain.
Chong NIM, et al. (2021, Singapore) [78]	Descriptive	Detail the implementation of CRT within an early psychosis intervention service in Asia and assess its impact on individuals with FEP by comparing cognitive assessment scores before and after CRT.
Drake, et al. (2014, UK) [91]	Naturalistic RCT	Evaluate whether administering CR before CBTp enhances the efficacy of CBTp in reducing delusions and hallucinations and investigate whether CR before CBTp allows CBTp to be completed more quickly or enables greater progress before completion, thus enhancing the efficiency of CBTp.
Medella, et al. (2015, Canada) [60]	Pilot RCT	Pilot test a standardised CCT intervention with individuals experiencing their first episode of schizophrenia.
Ventura, et al. (2017, USA) [93]	RCT	Investigate the potential benefits of CR on secondary, non-targeted areas such as negative symptoms and social functioning within the framework of a psychiatric rehabilitation programme.

^1^ COPE—cognitively oriented psychotherapy for early psychosis; ^2^ CBT/CBTp—cognitive—behavioural therapy/CBT for psychosis; ^3^ TAU—treatment as usual; ^4^ FEP—first-episode psychosis; ^5^ BDNF—brain-derived neurotrophic factor; ^6^ ICR—integrated cognitive remediation; ^7^ SCIT—social cognition and interaction training; ^8^ NEAR—Neurocognitive Educational Approach to Remediation; ^9^ CCT—compensatory cognitive training; ^10^ FMSG—Family-Led Mutual Support Group; ^11^ PANSS—Positive and Negative Syndrome Scale; ^12^ CR/CRT—cognitive remediation/cognitive remediation therapy; ^13^ SA—Social anxiety; ^14^ SM—symptom management; ^15^ Pr-EP—Parma-Early Psychosis programme; ^16^ MOL—Method of Levels; ^17^ SocialMIND—mindfulness-based social cognition training; ^18^ PMI—psychoeducational multicomponent intervention.

## Appendix C

**Table A3:** Participant characterisation *.

Diagnosis/Age/Target
[56] Early psychosis/[17–34 years]/Patient
[49] FEP/[15–29 years]/Patient
[92] FEP/[15 to 40 years]/Patient/family
[19] FEP/[18 to 45 years]/Patient
[73] FEP/[18 to 54 years]/Patient/family
[50] Carer of individuals with FEP/[carer role < 3 years of FEP patients [15–25 years]/Carer
[68] FEP/[18 to 30 years]/Patient
[61] Recent-onset psychosis (≤5 years of illness)/[≥18 years]/Patient/family
[84] FEP/[>16 years]/Patient
[51] FEP/[15 to 25 years]/Patient
[85] Early-onset psychosis/[14–18 years]/Patient/family
[67] Early-onset psychosis/[14 to20 years]/Patient/family
[77] Early psychosis/[18 to 65 years]/Patient
[79] FEP (cannabis users)/[15 to 40 years]/Patient
[70] Early psychosis/[16 to 35 years]/Patient
[86] FEP/[16 to 45 years]/Patient
[64] First episode of schizophrenia-spectrum disorders/[not mentioned]/Patient
[65] FEP/[18 to 45 years]/Patient/family
[87] Recent onset schizophrenia/[14 to 22 years]/Patient
[57] FEP/[18 to 35 years]/Patient
[58] FEP/[18 to 35 years]/Patient/family
[63] FEP/[late adolescent to young adult]/Patient/family
[69] Carers of persons with FEP/[Carers > 18 years]/Caregiver
[62] Carers of persons with FEP/[15 to 25 years]/family
[76] Ultra-high risk for developing psychosis or FEP/[16 to 65 years]/Patient
[74] FEP or at ultra-high risk for developing psychosis/[12 to 54 years]/Patient/family
[71] FEP/[Not mentioned]/Patient
[72] FEP/[Not mentioned]/Patient/family
[88] FEP/[16 to 35 years]/Patient
[89] FEP/[16 to 65 years]/Patient
[80] FEP/[18 to 45 years]/Patient
[66] At-risk mental state or FEP/[16 to 30 years]/Patient/family
[52] FEP/No limitations/Family
[90] Carers of service users of FEP/[not mentioned]/Carer
[75] UHR or FEP/[13 to 18 years]/Patient/family
[53] FEP/[15 to 25 years]/Patient/family
[54] Early psychosis/[15 to 29 years]/Patient
[81] Early-onset psychosis/[14 to 18 years]/Patient/family
[31] Recent onset of psychosis/[17 to 26 years]/Patient/family
[55] Carers of persons with FEP/[Not mentioned]/family
[83] FEP/[18 to 35 years]/Patient/family
[59] FEP/[16 to 50 years]/Patient/family
[82] FEP/[14 to 40 years]/Patient/family
[78] FEP/[12 to 40 years]/Patient
[91] First episode of non-affective psychosis/[18 to 35 years]/Patient
[60] Primary psychotic disorder/[18 to 35]/Patient
[93] Recent onset of psychosis/[18 to 45 years]/Patient

* Description of participant characterisation, in terms of diagnosis, age, and target.

## Appendix D

**Table A4:** Programme characterisation/implementation context.

Ref.	Frequency	Strategy/Content	Evaluation	Intervention Facilitators	Implementation Context
	Number of Sessions (NS) Treatment Duration (TD) Duration of Sessions (DS) Frequency of Sessions (FS) Follow Up (FU)				Setting Individual/Group
[50]	NS—5 sessions TD—5 weeks DS—2 h per session FS—1 module per week FU—6-week and 16 weeks	The intervention comprises a self-help manual entitled *Reaching Out: Supporting a Family Member or Friend with First-Episode Psychosis*. The manual, based on problem-solving therapy, is divided into modules that may be completed independently by carers. The objective is to improve the well-being of carers and enhance their caregiving abilities. The content covers a range of topics, including improving physical and mental health, developing strategies to access support services, supporting the well-being of the person with FEP, and managing the effects of the illness. The modules provide guidance on addressing communication challenges, lack of motivation, social withdrawal, risky behaviours, sleep disturbances, hallucinations, delusions, weight gain, medication adherence, substance misuse, aggression, and suicidal behaviour. To support the implementation of the material, a research officer conducts weekly telephone calls to discuss specific modules and clarify any questions. This intervention aims to empower carers by equipping them with the knowledge and practical skills necessary to manage their caregiving role while maintaining their well-being.	Experience of Caregiving Inventory (ECI) Kessler Psychological Distress Scale (K10) Family Questionnaire (FQ) Short-Form Health Survey (SF-12)	Not applicable	Urban zone Home-based setting TAU—individual and group session
[55]	NS—5 sessions + 5 telephone calls TD—5 weeks DS—2 h/session + 10 min telephone calls/week FS—Weekly FU—6- and 16-week follow-up	The intervention involved five modules, each requiring up to 2 h to complete, consisting of reading and exercise materials. The modules were as follows: (1) strengthening carer well-being and coping skills; (2) getting the best out of support services; (3) promoting the well-being of the person with FEP, focusing on preventing relapse and understanding treatment; (4) dealing with the effects of the illness Part A, which included communication, lack of motivation, social withdrawal, risky and unrestrained behaviour, disturbed sleep, hallucinations, and delusions; and (5) dealing with the effects of the illness Part B, covering issues such as weight gain, reluctance to take medication, substance misuse, aggression, and suicidal behaviour. Carers completed all modules independently. Research officers were trained to follow a standardized procedure for communicating with participants and collecting data. To monitor treatment adherence, a research officer conducted weekly 10 min telephone calls, asking standardized questions about the content of specific modules. These calls also provided an opportunity for participants to clarify any material from the modules.	Social Problem-Solving Inventory—Revised Short Form (SPSI-R:S)	Training research officers	Outpatient Individual
[81]	NS—three individual sessions (families and adolescents separately) + twelve group sessions TD—not mentioned DS—50 min/individual sessions; 90 min/group sessions FS—bi-weekly FU—2 years	The psychoeducational intervention involves running two simultaneous and parallel groups: one for parents and the other for adolescents. The therapy is divided into two main phases, adapted from W. McFarlane’s model: the initiation/alliance phase and the group phase, following a Multifamily Therapy (MFT) format. During the initiation phase, three individual sessions are conducted separately for families and adolescents. The group phase consists of twelve group sessions for patients and parents, focusing on problem-solving strategies to manage daily life difficulties related to the disease, mitigate crises, and prevent relapses. Written psychoeducational material is provided to both patients and families. The structure of each group session includes the following components: Informal Talk (10 min): A social conversation aimed at building alliances and social networks, avoiding discussing problems. Task Review (5 min): The family targeted in the previous session reviews the implementation of suggested solutions. Seminar (10 min): A brief presentation by a group leader summarizing a specific educational topic. Word Round (15 min): An informal discussion where all members can share their difficulties or concerns. Troubleshooting (40 min): Leaders select a dilemma or conflict for group discussion. Members brainstorm solutions, which are recorded and analysed. The session ends with an action plan agreed upon by the group, based on the preferred solution of the family or patient involved. Social and Informal Chat (10 min): Participants engage in conversations about hobbies or personal interests, intentionally avoiding problem-related topics.	Affective Disorders and Schizophrenia for School-Age Children—Present and Lifetime version (K-SADSPL) PANSS Children’s Global Assessment Scale (C-GAS)	Each group consists of two clinicians who are required to have a basic understanding of psychosis. Therapists are given feedback and are supervised by both team members and an external consultant. Weekly supervision sessions, lasting 1.5 to 2 h, are held with the full team. These sessions focus on maintaining adherence to techniques, enhancing the therapists’ skills, training new therapists, providing ongoing education, and resolving any clinical challenges that arise.	Outpatient setting Individual and group sessions (between 8 and 12 participants)
[86]	NS: - Group CRT: 2–3 times weekly for 14 weeks. - Independent CRT: Up to 41 sessions. - Intensive CRT: Twice weekly for 10.5 weeks. TD: - Group CRT: 14 weeks. - Independent CRT: 14 weeks. - Intensive CRT: 10.5 weeks. DS: - Group CRT: 90 min per session. - Independent CRT: 60–180 min per session. - Intensive CRT: 60–180 min per session. FS: - Group CRT: 2–3 times per week. - Independent CRT: Fortnightly contact. - Intensive CRT: Twice weekly. FU: 39 weeks post-randomization (±4 weeks window).	The intervention utilizes the CIRCuiTS computerized CRT programme, implemented through three different delivery modes: intensive CRT, group CRT, and independent CRT. Each mode varies in the amount of therapist contact but offers the same total treatment hours. Intensive CRT: This mode includes three components: (1) therapy sessions with a therapist, (2) in-vivo transfer work where CRT strategies are applied to real-life situations with therapist support, and (3) independent CRT activities set up by the therapist, either on-site or off-site during the participant’s own time. Group CRT: Participants engage in group therapy sessions with a closed membership of four participants per group, led by one therapist. Sessions start and end with group activities focused on goal setting and metacognition. During the session, participants independently work on CIRCuiTS tasks, with the therapist available to provide assistance as needed. Independent CRT: This mode involves an initial individual session with a therapist for orientation, followed by sessions where participants work independently. Support for these independent sessions includes telephone contact or drop-in sessions, available on an as-needed basis but limited to a maximum of one hour of contact time per fortnight. Sessions are considered valid if they last at least 20 min.	Goal Attainment Scale (GAS) Time Use Survey EuroQol 5-Dimensions 5-Levels (EQ-5D-5L) Client Service Receipt Inventory Rosenberg Self-Esteem Scale Cambridge Neuropsychological Test Automated Battery: (Reaction Time, One-Touch Stockings of Cambridge, Paired-Associates Learning, Attention Switching Task, Rapid Visual Information Processing, Spatial Working Memory and Emotion Recognition Task) and supplemented by the Computerized Wisconsin Card Sorting Task) Rey Auditory Verbal Learning Test Rey Osterrieth Complex Figure Digit Span forwards and back test Wechsler Abbreviated Scale of Intelligence	Therapy at each site is administered by an experienced assistant psychologist, trained in CRT at the trial centre and receiving weekly central supervision. Each therapist delivers all three types of CRT throughout the therapy period.	Individual/Group (4 participants/group) Outpatient
[87]	NS: Not specified TD: 3 months (40 h) DS: Not specified FS: 3 sessions/week. FU: 3 months (post-therapy)	The intervention involves a structured cognitive rehabilitation programme designed to enhance memory, complex planning, and problem-solving skills through graded tasks and systematic training. The programme consists of the following key components: Therapist Demonstration: The therapist explicitly demonstrates information processing strategies. Overt Practice: Participants practised these strategies openly under the therapist’s guidance. Covert Practice: As proficiency increases, participants progress to using these strategies covertly. Responsibilities: Participants engage in progressively challenging tasks aimed at developing cognitive skills. They apply information processing strategies and organisational techniques. Errors are minimised through regulated and monitored task execution. Participants work towards increasing their independent use of cognitive strategies over time. Supervision and Monitoring: Therapist adherence to the intervention protocol is ensured through direct observation. Regular supervision sessions are conducted to provide continuous support and ensure fidelity to the programme.	Wisconsin Card Sort Test (WCST) Wechsler Adult Intelligence Scale-III (WAIS-III-UK) Modified Six Elements Test Social Behaviour Schedule—total problem score on SBS Brief Psychiatric Rating Scale Quality of Life Scale Rosenberg Self-Esteem Scale	Not mentioned	Inpatient and community Individual
[78]	NS: 24 sessions TD: Total of 36 h (3 months) DS: Each session lasting 1.5 h FS: Twice a week FU: No follow-up	CRT: This intervention integrates a NEAR with the Cogpack software to improve cognitive abilities. NEAR Approach: Group Sessions: These sessions are designed to promote social interaction and consolidate learning. Focus: Emphasises setting both short- and long-term goals, encouraging patients to develop insight and motivation. Socio-emotional Context: Stresses the importance of understanding the socio-emotional environment and its effects on cognitive performance. Cogpack Software: Exercises: Features 64 structured neurocognitive tasks, classified into domain-specific and non-domain-specific categories. Domain-Specific Exercises: Address specific cognitive functions, including verbal memory, fluency, motor coordination, attention (sustained and selective), working memory, and executive functions. Non-Domain-Specific Exercises: Involve tasks that engage multiple cognitive domains, such as language skills, cultural knowledge, and basic logical and mathematical reasoning. Adaptive Difficulty: The software adjusts the difficulty of exercises based on the patient’s performance to ensure tasks remain suitably challenging. Occupational Therapy (OT) Involvement: Occupational therapists choose exercises that target specific cognitive areas and tailor sessions according to each patient’s progress and needs. Cultural Relevance: Exercises not relevant to the Singapore context are skipped. CRT Implementation Starting Point: Begins with repetitive drilling exercises using Cogpack. Progress Tracking: Cogpack displays results after each exercise, showing patient performance. Encouragement: Patients receive support and motivation from OTs. Learning Facilitation: New words related to pictures are taught to promote learning and recall. Hierarchical Structure: Exercises start with attention, processing speed, and reaction, then progress to memory and problem-solving. NEAR Principles and Games Positive Learning Experience: Ensures clients have a positive attitude about learning. Optimal Cognitive Functioning: Games promote optimal cognitive functioning. Examples of NEAR Games: Carmen Sandiego, Hot Dog Stand, and Fripple Place. Bridging Sessions Discussion: Patients discuss games and strategies used. Contextualization: Strategies are contextualized to real-life situations and roles. Social Interaction: Facilitates social interaction among patients and OTs. Skill Transferability: Enhances the transfer of skills learned in sessions to real life. Progress and Feedback Self-Recording: Patients record their progress, noting activities and performance. Goal Setting: Patients set targets to motivate subsequent sessions. Monthly Reviews: Individual review sessions are conducted to: Highlight patient progress Gather feedback on patient feelings and session goals Adjust treatment plans as needed	Montreal Cognitive Assessment (MoCA) Brief Assessment of Cognition in Schizophrenia (BACS) PANSS Global Assessment of Functioning (GAF)	Not mentioned	Group Maximum five clients/session. Outpatient
[60]	NS: 12 sessions TD: 24 h DS: 2-h group treatment session FS: Weekly FU: After 12 weeks, each participant was re-administered the outcome measures	CCT: This brief, group-based intervention addresses cognition in four key areas: prospective memory, attention and vigilance, learning and memory, and executive functioning. The training uses interactive, game-like activities to maintain engagement and enhance focus and motivation. Session Structure: Review: Discuss the previous week’s home exercises. Rationale: Explain the purpose of the new skills to be taught. Demonstration: Show how to perform the skill. Practice: Allow participants to practice the skill. Implementation: Plan how to apply the skill in daily life. Home Exercises: Assign tasks for the upcoming week to reinforce learning and develop cognitive habits. (CCT manual is available for free at www.cogsmart.com).	Wechsler Test of Adult Reading (WTAR) Measurement and Treatment Research to Improve Cognition in Schizophrenia Consensus Cognitive Battery (MCCB) University of California, Performance Based Skill Assessment—Brief Version (UPSA-B) PANSS Calgary Depression Scale for Schizophrenia (CDSS)	Not Mentioned	Group intervention 7–8 participants per group Outpatient
[93]	NS: 48 sessions TD: 12 months DS: 2 h (6 months), 1 h (3 months), 1 h (3 months) FS: Weekly FU: Not mentioned	Cognitive Remediation Programme: Part of the UCLA Aftercare Programme, this intervention combines computerized cognitive training with a Bridging Group to enhance generalization in psychiatric rehabilitation. Training Components: Software Programms: Utilises 23 computer-based programms adapted from brain injury rehabilitation (Bracy, 1994) and integrated into Neurocognitive Enhancement Therapy (NET) (Bell et al., 2007) and the NEAR for children and adolescents (Medalia and Revheim, 1999). Exercises: Designed with escalating difficulty levels, conducted in a computer lab with group sessions at the clinic. Learning Lab: Cognitive Coaches: Assist patients by reinforcing positive cognitive strategies, recommending methods to enhance cognitive skills, and monitoring patient progress.	Scale for the Assessment of Negative Symptoms (SANS) Brief Psychiatric Rating Scale (BPRS) UCLA Social Attainment Survey (SAS)	Trained cognitive coaches deliver the cognitive training using a manualised approach based on the NEAR principles (Medalia et al., 2009).	Urban Outpatient Individual
[89]	NS: No minimum or maximum number of sessions TD: 10 months DS: Not mentioned FS: Sessions were routinely available on 2 days of the week during working hours (User-led appointment scheduling) FU: Follow-up assessments at 10 and 14 months	Transdiagnostic Cognitive Therapy: Based on Perceptual Control Theory (Powers, 2005), this approach addresses goal conflicts and supports the reorganization process through the Method of Levels (MOL). MOL: Sessions: Therapists facilitate sessions where individuals discuss whatever is on their mind, focusing on “disruptions”—brief shifts in awareness. The therapist encourages elaboration on these moments to explore underlying issues. Consistent Principles: Unified Approach: The core principles of MOL remain consistent across different issues, streamlining the training and supervision of professionals. User-Determined Focus: Tailored Therapy: The therapy is customised to address the individual’s specific needs, with the focus of the sessions guided by the service user. User-Led Scheduling: Enhanced Autonomy: Service users have significant control over the timing and content of their therapy sessions, promoting greater engagement and self-direction.	Psychological Outcome Profiles (PSYCHLOPS) Clinical Outcomes in Routine Evaluation—Outcome Measure (CORE-OM) Reorganisation of Conflict Scale (ROC) Questionnaire about the Process of Recovery (QPR) Outcome Rating Scale (ORS) Session Rating Scale (SRS)	MOL sessions were conducted by the first author, a mental health nurse with extensive experience in delivering psychological interventions within early intervention services and postgraduate training in CBT for psychosis.	Individual Outpatient
[80]	NS: 17 sessions TD: 36 weeks DS: 90 min FS: 8 weekly sessions, 4 bi-weekly sessions, 5 monthly sessions FU: Not mentioned	Programme Overview: The intervention integrates formal meditation practices tailored for individuals with psychosis and social cognition exercises inspired by SCIT. It utilises mindfulness approaches including the Mindfulness-Based Stress Reduction (MBSR) programme, Mindfulness-Based Cognitive Therapy (MBCT), and the Mindful Self-Compassion (MSC) programme. Session Structure: Session 1: Present Moment Awareness Welcome and group introduction: Establishing guidelines Introduction to mindfulness principles Session 2: Perception Diversity Distinguishing between interpreting and describing experiences Encouraging a pause before making assumptions about others’ motivations Session 3: Managing Distress Understanding human responses to pleasant, unpleasant, and neutral experiences Addressing the tendency to avoid unpleasant experiences Session 4: Radical Acceptance Accepting all experiences without resignation Recognising mental events as products of the mind Session 5: Unconditional Friendship and Compassion Promoting well-being through self-compassion and loving-kindness Raising awareness of self-criticism and judgmental attitudes Session 6: Cultivating the Wholesome Seeking pleasant experiences to counterbalance negative biases Session 7: Relationships and Connection Emphasising safe interaction and connection with others Introducing mindful dialogue and pausing during tension Session 8: Balanced Living Practising equanimity with both pleasant and unpleasant experiences Completing the 8-week SocialMind training and awarding certificates Sessions 9–12: Consolidation Interactive sessions focusing on personal choices, interpersonal practice, the STOP technique, and cultivating equanimity and compassion Encouraging awareness of cognitive distortions Sessions 12–15: Integration Continued interactive sessions aimed at integrating learned skills into daily life, with a focus on interpersonal practice and mindfulness techniques	Personal and Social Performance (PSP) General Assessment of Functioning scale (GAF) PANSS Calgary Depression Scale for Schizophrenia (CDSS) self-reported Beck Anxiety Inventory (BAI) Clinical Global Impression for Bipolar Disorder (CGI-BD) 5-item version of the Hinting Task Eyes Test Penn Emotion Recognition Test (ER-40) Ambiguous Intentions Hostility Questionnaire (AIHQ) Mindful Attention and Awareness Scale (MAAS) Matrics Consensus Cognitive Battery (MCCB)	SocialMIND teachers are certified teachers of these programmes	Outpatient Groups are composed of a maximum of 15 participants
[62]	NS: 6 TD: 6 months DS: 1.5 h/session FS: Weekly FU: 6 weeks after group therapy sessions, and 6 months after completion of the session	Caregiver Education Programme: This programme focuses on equipping caregivers with essential knowledge and skills related to managing early psychosis and supporting individuals through effective strategies. Key Topics: Education on Early Psychosis and Treatment: Understanding the early signs of psychosis and available treatment options. Handling Difficult Behaviours: Techniques for managing challenging behaviours associated with psychosis. Stress Management: Skills to cope with the stress of caregiving and managing psychosis. Communication Skills: Enhancing strategies for effective interaction with individuals experiencing psychosis. Relapse Prevention: Methods for recognizing early signs of relapse and strategies to prevent recurrence. Session Structure: Each session commenced with a social period and a review of weekly events or progress on assignments. Participants were encouraged to pose questions and suggest discussion topics. A 15 min break was included to facilitate interpersonal interaction. Caregivers involved in the study did not receive intensive individual or family psychotherapy, except those in the active intervention group. Continued engagement with the patient’s case manager, typically a psychiatric nurse or medical social worker, was maintained as per protocol.	PANSS Knowledge about psychosis scale Experience of Caregiving Inventory (ECI) Chinese Ways of Coping Questionnaire (CWCQ) Level of expressed emotion (LEE) General Health Questionnaire (GHQ)-12 Life Events Questionnaire	Masters-level psychologist (under the supervision of the first author).	Outpatient Group intervention (8 groups of 4 to 8 each)
[52]	NS: 4 TD: 1 month DS: 2 h/session FS: One night a week FU: No follow-up	4-Week Programme Overview: This programme offers a structured approach to understanding and managing psychosis. Session Breakdown: Session 1: Introduction to Psychosis Overview of the programme: Definition and exploration of psychosis in terms of thoughts, feelings, and behaviours. Discussion on diagnosing psychosis and potential causes. Gathering participant goals and feedback. Session 2: Biological Treatments Overview of major medication groups: antipsychotics, antidepressants, mood stabilisers, and sedatives. Discussion of non-biological treatments. Session 3: Stress and Psychosis Examination of the stress-vulnerability model. Exploration of stress impacts and communication styles. Introduction of problem-solving strategies. Session 4: Relapse Prevention and Support Focus on identifying early warning signs and relapse prevention. Overview of mental health services and community support. Access to written materials and videos. Session Structure: Each session includes a break for interaction and private questions. Content for the next session is briefly introduced at the end of each session. Weekly reviews start each session, revisiting the previous week’s content. Evaluation: Formal and informal evaluations through discussion and questioning. Addressing issues related to grief, community attitudes, and support needs as they arise.	Not mentioned	Two community mental health nurse clinicians facilitate the groups and undergo training as outlined by Laube and Higson (2000).	Community Group (no designated number for Each group and no rules regarding having a closed group)
[90]	NS: 3 TD: 3 weeks DS: 2 h/session FS: Weekly FU: No follow-up	Programme Overview: This cognitively focused programme operates within a bio-psycho-social framework, addressing the onset, maintenance, and relapse of psychotic disorders. Session Breakdown: Session 1: Understanding Psychotic Conditions Discusses symptoms, causes, progression, timelines, impact, and available treatments for psychotic disorders. Session 2: Caregiver Support Emphasizes adaptive coping strategies for caregivers, focusing on their experiences and needs. Session 3: Approaches to Caregiving Explores various caregiving approaches, addressing specific challenges and problems faced by caregivers. Session Structure: Consistent agenda for each session. Opportunities for peer-to-peer discussion. Homework assignments to reinforce learning. Focus on linking beliefs, emotions, and behaviours to adaptive coping strategies. Delivery of Content: Content was delivered via PowerPoint presentations and facilitated group discussions. Handouts from workshops were provided to group members.	Adapted illness belief questionnaires.	Consultant psychiatrist and clinical psychologist from the psychosis team	Community Group
[88]	NS: Maximum of 26 sessions TD: 6 months DS: Not mentioned FS: Weekly FU: 6 months (post-treatment) and 12 months follow-up	CR Intervention: CR intervention includes three main components: Engagement and Formulation: Builds a therapeutic relationship and understands the individual’s issues. Trauma Processing: Tailored for trauma-related issues, this explores initial psychotic episodes, symptoms, management, and social context using the “Back in the Saddle” framework (Plaistow and Birchwood, 1996) for relapse prevention. Appraisals of Psychotic Illness: Examines perceptions of psychotic experiences within social rank theory (Gilbert and Allen, 1998) using cognitive therapy techniques such as Socratic questioning, guided discovery, targeting beliefs and behaviours, developing alternative beliefs, and reinforcing new beliefs through behavioural change.	The Impact of Events Scale The Calgary Depression Scale (CDSS) The Robson Self Esteem Questionnaire (SCQ)	The CR was delivered as per the protocol by four clinical psychologists and a cognitive–behavioural psychotherapist. All clinicians had over four years of experience in cognitive therapy for early psychosis and received regular case supervision.	Urban Individual Outpatient
[54]	NS: 8–10 sessions TD: 10 weeks DS: Not mentioned FS: Not mentioned FU: Follow-up at 6 months	LifeSPAN Therapy: This brief, individual cognitive-oriented therapy includes four key phases: Initial Engagement: Focuses on detailed, collaborative risk assessment and formulation of suicidality to identify key areas for intervention in subsequent phases. Suicide Risk Assessment/Formulation: This phase involves a thorough evaluation of suicide risk, including identifying and understanding the factors contributing to suicidality. Cognitive Modules: Consists of eight modules covering: Core Module: Functional analysis of suicidality, reasons for suicide, hopelessness, and reasons for living. Additional Modules: Problem-solving training, psychoeducation for psychosis, emotional pain tolerance, stress management, self-esteem enhancement, help-seeking, and social skills training. Final Closure/Handover: The therapist and patient review and confirm the suicidality formulation. Identification of early warning signs and triggers for suicidal ideation or behaviours. Reinforcement of suicide-protective strategies, including support for self-esteem and help-seeking options. The patient’s case manager participates in the final session to develop a Care Plan for ongoing risk management and prevention.	Brief Psychiatric Rating Scale (BPRS) (Expanded Version 4) Suicide Ideation Questionnaire (SIQ) Suicide Intent Scale Reasons for Living Inventory (RFL-24) Scale for the Assessment of Negative Symptoms (SANS) Quality of Life Scale Global Assessment of Function (GAF) Scale Beck Hopelessness Scale (BHS) Self Esteem Scale (SES) Self-Report Problem-Solving Rating Scale (SRPSRS)	The programme employed 2.5 staff members, including two full-time clinical psychologists, at MHSKY (Mental Health Services for Kids and Youth). Therapy was provided by one of these two psychologists, independent of the EPPIC service.	Mix urban/rural Outpatient setting Individual sessions
[51]	NS: Maximum of 20 sessions of therapy TD: 14 weeks (no more than 2 weeks past the 12-week assessment) DS: ±45 min/session; flexible FS: Frequency flexible, depending on participant needs FU: 1-year follow-up	Active Cognitive Therapy for Early Psychosis (ACE): This CBT approach involves: Assessment: Evaluates both psychotic and non-psychotic complaints, along with their relationship to the participant’s life history. Formulation and Prioritisation: Uses a flowchart to prioritize treatment areas, focusing on: Risk Issues: Addressed as a priority. Positive Psychotic Symptoms: If distressing and present. Co-morbidities: Managing additional disorders. Negative Symptoms: Addressing impairments in motivation and emotion. Issues of Identity and Relapse Prevention: Ensuring long-term stability. Treatment Approach: Each identified difficulty is addressed from a broadly cognitive–behavioural perspective.	Psychotic Subscale of the Brief Psychiatric Rating Scale (BPRS) Assessment of Negative Symptoms (SANS) Social and Occupational Functioning Assessment Scale (SOFAS) Cognitive Therapy Rating Scale (CTRS)	Two clinical psychologists (E.K., S.B.) delivered both treatments. The therapists received three months of training in the treatments and were supervised throughout the trial.	Urban Home based Individual
[67]	NS: 20 individual sessions TD: 9 months DS: Not mentioned FS: First four sessions weekly, 16 sessions fortnightly; timing flexible based on client needs FU: 24-month follow-up	CBT: Initial Psychoeducation: The first three sessions focused on psychoeducation, conducted early in the therapy to provide foundational knowledge. Relapse Prevention: The final two sessions, scheduled towards the end of therapy, concentrated on strategies for relapse prevention. Module-Based Approach: Post-assessment and engagement, the therapy followed a manual with modules targeting various symptom areas, including delusions, hallucinations, negative symptoms, and comorbid conditions. Stabilization and Relapse Prevention: Offered towards the end of treatment to consolidate gains and prepare for future challenges. Optional Caregiver Involvement: If available, five optional sessions were provided for parents or caregivers, with the final session including both caregivers and the patient.	Positive and Negative Syndrome Scale from PANSS Psychotic Symptoms Rating Scales (PSYRATS) Calgary Depression Scale for Schizophrenia (CDSS) Global Assessment of Functioning scale (GAF) Modular System for Quality of Life (MSQoL-R)	Four clinical psychologists, all in advanced CBT training, provided therapy. They received specific training in the manual’s application and had a high level of expertise in treating psychotic disorders. Although formal assessments of competence and adherence to the treatment manual were not conducted, therapists were regularly supervised by the PI (A.B.) and local PIs (P.W., G.L., K.M., J.H., G.W., D.S., S.K.), in addition to peer supervision.	Urban Outpatient Individual
[77]	NS: 26 sessions TD: 6 months DS: 45–60 min FS: Weekly FU: 15-month follow-up	Stage 1 (Sessions 1–5): Focuses on engagement, introducing CBT principles and the stress–vulnerability model, and setting expectations. It emphasises active participation, collaboration, homework, and developing a problem list and goals. Stage 2 (Sessions 6–20): Targets depressive symptoms and low self-esteem, the main focus of the study. It involves creating a problem list to address everyday psychological challenges and using Morrison’s case formulation, which is updated throughout therapy. Stage 3 (Sessions 20–26): Dedicated to therapy termination and relapse prevention, summarising therapy, and helping the patient become “their own cognitive therapist.”	PANSS Calgary Depression Scale for Schizophrenia (CDSS) Beck Depression Inventory (BDI-II) Rosenberg Self-Esteem Scale (RSES) Premorbid Assessment Scale (PAS) Global Assessment of Functioning (GAF) Alcohol Use Disorder Identification Test (AUDIT) Drug Use Disorders Identification Test (DUDIT)	A dedicated CBT treatment team included two clinical psychologists (one female, one male), two psychiatrists (one female, one male), and an occupational therapist (female). All therapists completed a two-year educational programme in CBT provided by The Norwegian Association of Cognitive Therapy. Additionally, they attended monthly meetings starting two years before the study baseline to learn and practice the specific CBT manual used in the study.	Outpatient Individual
[71]	NS: 12 group sessions TD: 12 weeks DS: 1 + 1/2 h per session FS: Weekly FU: Booster session 3–4 months	CBT for Psychosis: This intervention aimed to empower participants through psychoeducation, normalization, and anxiety management, while disempowering psychotic symptoms using cognitive restructuring and mindfulness techniques. The treatment was tailored separately for FEP and schizophrenia (SP) groups. Personal issues were addressed individually at the end of sessions, providing necessary support while not focusing on specific conditions like trauma.	Scale for the Assessment of Negative Symptoms (SANS) Scale for the Assessment of Positive Symptoms (SAPS) Calgary Depression Scale (CDS) World Health Organization Quality of Life, Brief Version (WHO QOL-Bref) Worry subscale of the Somatic, Cognitive and Behaviour Anxiety Inventory Wechsler Abbreviated Scale for Intelligence (WASI)	Not mentioned	Community-based, urban region Group sessions
[79]	NS: 16 sessions TD: 16 weeks DS: 1 h per session FS: Weekly FU: 3 and 6 months, and 1 year follow-up from the end of the intervention programme	Cognitive–Behavioural Therapy for Cannabis Cessation with Pharmacological Treatment: This programme focuses on cannabis cessation, recognizing prodromes, enhancing illness awareness, ensuring treatment adherence, improving psychosocial functioning, and preventing relapse. Sessions 1–3: Begin with motivational interviewing and brief psychoeducation covering topics such as the relationship between psychosis and substance use, medication adherence, awareness of vulnerability, symptom recognition, healthy lifestyle, and risk and protective factors. Sessions 4–8: Emphasize commitment to change and include: Behavioural Therapy: Techniques for anxiety management, stimulus control, in vivo exposure with response prevention, identifying triggers and beliefs leading to substance use, and exposure to these triggers. Cognitive Therapy: Methods for managing thoughts related to cannabis use and cravings, cognitive restructuring, problem solving, social skills training, assertiveness, refusal skills, and lifestyle changes. Sessions 10–12: Focus on relapse prevention by identifying high-risk situations, teaching coping skills, and addressing factors that could lead to continued substance use and increased psychotic symptoms.	European Addiction Severity Index (EUROP-ASI) Cannabis Use Problems Identification Test (CUPIT) Clinical Global Impression Scale (CGI) Scale to assess Unawareness in Mental Disorders (SUMD) 4-item Morisky Medication Adherence Scale PANSS Hamilton Depression Rating Scale (HDRS-21) Young Mania Rating Scale (YMRS) Hamilton Anxiety Scale (HAM-A) Functioning Assessment Short Test (FAST)	Not mentioned	Outpatient Individual
[57]	NS: 13 group sessions TD: 13 weeks DS: 1.5 h per session FS: Weekly FU: Post-therapy, 3-month, and 6-month follow-ups	CBT for Social Anxiety (CBT-SA): This programme consists of five modules aimed at addressing social anxiety disorder, stress, psychosis, and self-stigma. Modules: Psychoeducation: Provides an understanding of SCIT disorder, stress, psychosis, and self-stigma. Cognitive Restructuring: Focuses on identifying and restructuring negative thoughts related to anxiety-provoking situations. Social Skills Training: Enhances interpersonal skills through structured practice sessions. Exposure Component: Involves collecting information to reassess judgments about perceived risks and challenging dysfunctional beliefs. Relapse Prevention and Maintenance: Develops strategies to prevent relapse and sustain progress. Responsibilities: Engage actively in all modules, apply techniques to real-life situations, participate in group discussions and exercises, and monitor progress while using relapse prevention strategies to maintain improvements.	The Social Interaction Anxiety Scale (SIAS) Social Phobia Inventory (SPIN) Brief Social Phobia Scale (BSPS) Scale for Assessment of Positive Symptoms (SAPS) Scale for Assessment of Negative Symptoms (SANS) Recovery Assessment Scale (RAS) Social and Occupational Functioning Scale (SOFAS) Calgary Depression Scale (CDS) Internalized Stigma of Mental Illness (ISMI) Wechsler Abbreviated Scale of Intelligence (WASI) Cognitive and Intelligence Assessment (CogState)	CBT-SA was delivered by a doctoral-level psychologist and a co-therapist using a group CBT-SA manual outlined by Montreuil et al. (2016). The intervention was supervised by an experienced CBT therapist (M.L.).	Mixed rural/urban Geographical diversity—Montreal Outpatient setting
[76]	NS: Not mentioned TD: 8 weeks (in addition to TAU) DS: Approximately 45–60 min per session FS: Not mentioned FU: 6-month and 12-month follow-ups	Acceptance and Commitment Therapy for Daily Living (ACT-DL): This intervention involves eight structured sessions conducted face-to-face by a trained clinician, including one session dedicated to psychoeducation. Participants then use the PsyMate™ smartphone app to apply learned skills in daily life. Sessions: First Six Sessions: Focus on six core ACT components tailored for psychosis: creative hopelessness, acceptance, cognitive diffusion, self-as-context, present moment awareness, values, and committed action. The final session reviews and integrates these components. ACT-Based Ecological Momentary Intervention (EMI): Starting from the second face-to-face session, participants use the PsyMate™ app to engage in EMI activities at least three days a week. The app prompts them at semi-random times to complete brief questionnaires on mood, psychotic experiences, and activities, providing ACT exercises or metaphors based on session content. The EMI progressively covers all ACT components to promote flexible application, including during distress. Completion: Participants’ access to the app ends after the intervention period.	Comprehensive Assessment of At-Risk Mental States (CAARMS) Global Assessment of Functioning (GAF) Social and Occupational Functioning Assessment Scale (SOFAS) Social Functioning Scale (SFS) Experience Sampling Method (ESM) on a smartphone-based app (the PsyMate™ app) Brief Psychiatric Rating Scale (BPRS) Brief Negative Symptom Scale (BNSS) PANSS	Trained clinicians (psychologists with a 5-day ACT-DL training and fortnightly supervision sessions)	Urban Individual
[84]	NS: 3 pseudo-randomized time points per day TD: 12 weeks DS: Not mentioned FS: Daily (6 days a week, between 10.00 and 22.00) FU: 12 and 22 weeks post-treatment	Acceptance and Commitment Therapy for Daily Living (ACT-DL): This intervention comprises eight face-to-face sessions led by a trained clinician, including a dedicated psychoeducation session. Following these sessions, participants utilize the PsyMate™ app to apply skills in daily life. Sessions: First Six Sessions: Focus on six core ACT components relevant to psychosis: creative hopelessness, acceptance, cognitive defusing, self-as-context, present moment awareness, values, and committed action. The final session integrates and reviews these components. ACT-Based Ecological Momentary Intervention (EMI): Beginning with the second session, participants use the PsyMate™ app for EMI activities at least three days a week. The app prompts users at semi-random times to complete brief questionnaires on mood, psychotic experiences, and activities, offering ACT exercises or metaphors related to session content. EMI covers all ACT components progressively to facilitate flexible application, including during distress. Completion: App access concludes after the intervention period. Actissist: A digital health intervention allowing spontaneous or prompted engagement. It collects user responses and uploads them to a server. Notifications: Persistent reminders on the handset until accepted, dismissed, or “snoozed” (up to 15 min). Users can also initiate the app at any time. Upon engagement, users select intervention domains and complete self-assessment questions on cognitive appraisals, beliefs, emotions, and behaviours. Based on the appraisal, users receive normalizing messages and coping strategies. Users can report “no problems like this” or interact self-initiatedly. Part 2: Offers multimedia options complementing intervention feedback, including relaxation exercises, recovery stories, fact sheets, external links, a daily diary, and emergency contacts. A graphical summary of the past week’s data aids in tracking distress and supporting self-management and treatment decisions. Target Domains: Auditory verbal hallucinations, paranoia, perceived criticism, socialization, and cannabis use.	PANSS Psychotic symptom rating scales (PSYRATS) Calgary Depression Scale for Schizophrenia (CDSS) Global Assessment of Functioning scale (GAF) Personal and Social Performance Scale (PSP) Empowerment Rating Scale (ERS) EuroQol 5-Dimensions 5-Levels (EQ-5D-5L) Timeline Follow Back (TLFB) Perceived criticism scale Medication Adherence Rating Scale (MARS)	Actissist is a standalone app that does not connect with external services.	Northwest of England Individual Actissist is standalone app that do not link with services. Individual
[56]	NS: 16 visits for ABCR; daily for CAT TD: 4 months DS: 1 to 2 h per visit; Computer 20 min daily FS: Weekly visits for CAT; not specified for ABCR FU: 5 months post-treatment	CAT (Cognitive Activation Therapy): This therapy provides environmental supports such as checklists, signs, and alarms, alongside compensatory strategies tailored to the individual’s environment, needs, and recovery goals. The approach involves differential support, ranging from structuring tasks and articulating steps to reducing distractions and enhancing organization based on an initial assessment of behavioural, environmental, and cognitive factors. ABCR (Advanced Brain Cognitive Rehabilitation): This programme features computerized cognitive exercises through 15 gamified tasks designed to improve attention, processing speed, visual memory, verbal memory, working memory, and executive functioning. The transition to real-world application involves practicing simulated work, social, recreational, and role-play tasks to integrate cognitive skills into everyday activities.	Multnomah Community Ability Scale (MCAS) Social and Occupational Functioning Scale (SOFAS) Social Functioning Scale (SFS) Goal Attainment Scaling (GAS) Brief Psychiatric Rating Scale (BPRS-E) Negative Symptom Assessment (NSA) The Wide Range Achievement Test (WRAT III; Wilkinson) The Trail Making Test Part A and Part B Weschler Adult Intelligence Scale (WAIS) California Verbal Learning Test Wisconsin Card Sorting Test (WCST) Digit Vigilance Test (DVT) Brief Adherence Rating Scale	CAT specialist	CAT + ABCR—home-based CAT—individual; ABCR—group-based (do not specify number of participants/group)
[70]	NS: 10 sessions (session 1–4 CRT; session 5–10 SRT) TD: 1 h DS: 1 h per session FS: Weekly FU: 12 weeks post-intervention	CRT Programme: Computerised Interactive Remediation of Cognition-Training for Schizophrenia (CIRCuiTS): This web-based CRT programme focuses on enhancing metacognition and cognitive functions such as attention, memory, and executive functioning through massed practice. The programme involves collaborative goal setting for real-world tasks, with exercises progressively increasing in difficulty based on participant performance. Initial weeks include remote practice sessions, which continue alongside in-person therapy. Social Recovery Therapy (SRT): Conducted from weeks 5 to 10 in conjunction with the CRT programme, SRT is informed by cognitive–behavioural theory and targets individual goals. It is delivered in three stages: Stage 1: Engagement and formulation to identify problems and establish a therapeutic relationship. Stage 2: Preparation for new activities, including identifying pathways and collaborating with community stakeholders. Stage 3: Engagement in new activities using behavioural experiments to encourage social interaction.	Intrinsic Motivation Inventory for Schizophrenia Research Social and Occupational Functional Assessment Scale (SOFAS) Time Use Survey Emotion Recognition Task (ERT) Hinting Task Bell Lysaker Emotion Recognition Task (BLERT) Reading the Mind in the Eyes Task Wechsler abbreviated scale of intelligence (WASI) Wechsler Memory scale (WMS) Rey Osterreith Complex Figure (ROCF) Stroop Colour and Word Test (STROOP) Need for Cognition Scale (NCS) PANSS	Online	Online
[64]	NS: 32 sessions TD: 16 weeks DS: 1 h per session (38 h total; mean weekly time—2.5 h) FS: Twice a week FU: Post-training assessments at 120 days; follow-up assessments at 300 days following the baseline assessment	The cognitive training programme was structured into four modules, targeting different cognitive domains. The first three modules addressed attention, executive functions, and learning/memory. The fourth module was customized based on the participant’s needs, determined through a combined evaluation with the trainer. Modules Overview: Modules 1–2: Focused on computer exercises for focused, divided, and sustained attention, as well as planning, strategy learning, and problem-solving. Tasks progressed in difficulty using COGNIsoft (http://www.cognisoft.dk). Module 2 (second half) and Module 3: Included practical everyday tasks such as meal preparation and compensatory training. Module 4: Offered a tailored combination of computer exercises and practical tasks based on the individual’s specific needs. Training Approach: Bottom-Up Approach: Emphasized repetitive drills and practice to enhance cognitive processing and automaticity. Top-Down Approach: Included strategy learning and guided problem-solving tailored to individual resources. Compensatory Strategies: Incorporated calendar training within the learning/memory module. Innovative Elements: Competence Dialogues: Semi-structured interviews conducted every other week aimed at maintaining motivation and bridging cognitive training with real-world skills. These dialogues focused on work competencies, self-experienced cognitive competencies, and social skills, facilitating the application of learned skills beyond cognitive exercises.	University of California, Performance Based Skill Assessment—Brief Version (UPSA-B) MATRICS Consensus Cognitive Battery (MCCB): speed of processing (BACS Symbol Coding, Category Fluency and Trail Making A), attention (Continuous Performance Test IP), working memory (Wechsler Memory ScaleIII, Letter–Number Sequencing), verbal learning—Hopkins verbal learning test-revised (HVLT-R), visual learning (Brief Visuospatial Memory Test—Revised), problem solving (Neuropsychological Assessment Battery, NAB Mazes), social cognition (Mayer–Salovey–Caruso Emotional Intelligence Test—MSCEIT) Trail-Making Test: Part B Hopkins Verbal Learning Test–Revised (HVLT–R) Danish Adult Reading Test (DART; i.e., Danish version of NART) PANSS The Rosenberg Self-Esteem Scale (RSE)	The cognitive trainers were psychologists and occupational therapists with professional psychiatric experience and a foundational knowledge of cognitive psychology.	Outpatient setting Individual basis
[49]	NS: 10 sessions TD: 12 months DS: 40 min per session FS: Weekly or fortnightly (flexible, depending on the patient’s mental state, phase of recovery, and availability) FU: After four years from the end of treatment	COPE is structured into four phases, though progression through these phases is flexible: Engagement: Initial phase focused on building rapport and setting the stage for therapy. Assessment: Lasts 3–4 sessions and involves creating an agenda or contract that includes psychoeducation, addressing stigma and identity issues, and tackling problems related to motivation and confidence. Adaptation: Focuses on adapting strategies to individual needs and circumstances. Secondary Morbidity: Addresses any secondary issues that arise, integrating solutions within the therapeutic framework. Phases are designed to be sequential but are not required to be followed in a fixed order.	Royal Park Multidiagnostic Instrument for Psychosis (RPMIP) Explanatory Model Scale (EM) Integration/Sealing Over (I/SO) measure Brief Psychiatric Rating Scale (BPRS) Psychotic Subscale of the BPRS Schedule for the Assessment of Negative Symptoms (SANS) 13-item Beck Depression Inventory (BDI) General Symptom Index (GSI) of the SCL-90-R Quality of Life Scale (QLS) Social and Occupational Functioning Assessment Scale (SOFAS)	Consultant psychiatrists and clinical psychologists receive weekly group and rotational peer supervision. The COPE therapist is not the treating medical doctor or case manager.	Patient Home-based Individual
[74]	NS: CBT: 10 sessions per patient Family Intervention: Minimum of 8 sessions per family Case Management: At least 20 sessions per patient in the first year TD: CBT: 3 months initially Family Intervention: First 6 months Case Management: 1 year initially, with subsequent ongoing sessions DS: CBT: Not specified Family Intervention: Not specified Case Management: Not specified FS: CBT: Weekly for the first 3 months, with booster sessions as needed Family Intervention: Sessions within the first 6 months, with additional booster sessions as needed Case Management: Weekly or as needed, with additional sessions based on progress and needs FU: CBT: Booster sessions as required Family Intervention: Booster sessions as needed - Case Management: 12 additional sessions per patient, with follow-up tailored to individual progress	The comprehensive intervention package integrates pharmacological treatment with a multi-component psychosocial intervention, including CBT-oriented individual psychotherapy, psychoeducational sessions for families, and recovery-oriented case management. After the 2-year intervention period of the Pr-EP protocol, patients and families may continue with treatment as usual, which includes pharmacological therapy and general case management support, without necessarily being discharged from mental health services. The Pr-EP involves four main processes: identification, assessment, intervention for FEP patients, and intervention for UHR individuals (such as those with Brief Limited Intermittent Psychotic Symptoms (BLIPS), Attenuated Psychotic Symptoms (APS), and Genetic Risk and Functional Decline (GRFD)). Each process includes specific procedures carried out in specialized mental health settings with defined durations, timings, and schedules. CBT-oriented individual psychotherapy follows the model developed by Fowler, Garety, and Kuipers (1995). For UHR individuals, an adapted model designed by van der Gaag et al. (2012) for psychosis-risk syndrome is utilized.	Not mentioned	Expert multi-professional teams, including psychiatrists, clinical psychologists, and case managers specializing in early intervention in psychosis (EIP), offer tailored treatments.Clinical psychologists delivering CBT-based individual psychotherapy undergo specific training programmes. Similarly, mental health professionals involved in family interventions, such as psychiatric nurses, educators, and psychiatric rehabilitation therapists, receive training in CBT-oriented psychoeducation.All mental health professionals participating in case management—psychiatric nurses, educators, social assistants, and psychiatric rehabilitation therapists—complete specific training programmes and undergo competence assessments. Detailed intervention manuals, based on international standards, guide the treatment process. A team of departmental experts supervises all interventions for subjects and their families, with monthly meetings and regular consultations to ensure continuous support. Additionally, Pr-EP professional teams meet bi-weekly to monitor individual care pathways. Throughout the 2-year intervention period, a minimum of five monitoring assessments (one every 6 months) are conducted, including re-administration of the Pr-EP assessment battery to evaluate progress and adjust treatment as needed.	Mixed urban/rural Community-based Not mentioned
[66]	NS: CM—3 times a week in a familiar environment; 5 times a week in community living TD: 2 years DS: 90 min per session FS: 3 times a week in familiar environment; 5 times a week in community living FU: Not specified	Hospitalization and Initial Contact: Patients who are hospitalized are met by at least one team member within 24 to 72 h. Non-hospitalized patients must be seen by the end of the week following referral. They are informed of their diagnosis and the 2-year treatment plan, with all FEP patients receiving low-dose antipsychotics. Case Management: Case managers facilitate access to treatment and collaborate with psychiatrists and psychologists, aiming to support recovery and socio-professional reintegration. They provide personal support, forming strong therapeutic alliances and integrating their presence into daily activities to help manage symptoms without hospitalization. Psychoeducative Approach: Case managers use a vulnerability–stress model to help patients develop personal skills and coping strategies. Patients learn to identify distressing situations and apply coping mechanisms such as avoidance, progressive confrontation, and stress management techniques. Individual and Group CBT: Patients receive both individual and group CBT. The first module covers hypothetical reasoning, stress management, self-esteem and mood, substance consumption, assertiveness, and communication skills. The second module focuses on developing assertiveness and communication skills through role-playing, with social functioning practiced in weekly group activities. Cognitive Remediation Therapy: Cognitive deficits are treated with computerized cognitive remediation using tools like Rehacom. The intensity and duration of treatment are tailored to the severity of deficits determined by neuropsychological evaluations. Substance Misuse Support: Patients with substance misuse issues are referred to addiction services, where they receive motivational interviews and may be hospitalized to aid in ceasing substance use. Systemic Therapy: Suggested to address family functioning disruptions, either with or without the patient. Monthly sessions with the psychiatrist and close relatives provide psychoeducative guidance on symptoms of FEP, daily functioning, and the vulnerability–stress model.	Comprehensive Assessment of At-Risk Mental State (CAARMS) PANSS Self-Evaluation of Negative Symptoms (SENS) Calgary Depression Scale (CDS) Young Mania Rating Scale (YMRS) World Health Organization Quality of Life, Brief Version (WHO QOL-Bref) Self-Esteem Rating Scale—Short Scale to Assess Unawareness of Mental Disorder (SUMD) Birchwood Insight Scale Global Assessment of Functioning (GAF) Premorbid Assessment Scale (PAS)	The team consists of 6 nurses/case managers, 1 psychiatrist, and 1 psychologist. Medical functions are decentralized, with the case manager serving as the pivotal team member, acting as the primary contact for each patient and coordinating medical and social support. All case managers are nurses, managing a caseload of 10 to 12 patients. They receive 1 week of training with case managers from the Treatment and Early Intervention in Psychosis Programme (Pr. Conus, Lausanne, Switzerland), aiming to acquire expert tools for both ambulatory and intensive care of FEP patients.	Outpatient Interventions are principally offered in the environment where the patient lives or spends the most time (eg, school, work or the library) on a case management model
[75]	NS: CBT: 20 sessions in the first year; 10 sessions in the second year; booster sessions from the third to the fifth year Family psychoeducation: 10 sessions Case management: 24 sessions in the first year; at least 50 additional sessions from the second to the fifth year TD: CBT: 12 months initially; ongoing up to 5 years with booster sessions Family psychoeducation: 12 months Case management: 12 months initially; ongoing up to 5 years DS: CBT: 1 h per session Family psychoeducation: Not specified Case management: 1 h per session FS: CBT: Weekly in the first year; less frequent in the second year; booster sessions as needed Family psychoeducation: Weekly or as scheduled within the first 12 months Case management: Weekly in the first year; frequency varies in subsequent years FU: Not specified	Intervention Components: The multi-element psychosocial intervention includes individual CBT, psychoeducational sessions for family members, and case management, following modern guidelines (NICE, 2013; RER, 2016; Schmidt et al., 2015). CBT details were not specified. Family Psychoeducation: The psychoeducational treatment consists of six main modules: (a) psychotic symptoms, (b) vulnerability–stress coping model, (c) substance abuse and psychosis, (d) medication and psychosis, (e) expressed emotion, and (f) stigma and recovery (Pelizza et al., 2019c). Booster sessions are provided as needed based on symptomatic areas and functioning. Case Management: A case manager coordinates all interventions, focusing on early recovery and rehabilitation.	Comprehensive Assessment of At-Risk Mental States (CAARMS) Social and Occupational Functioning Assessment Scale (SOFAS)	ReARMS teams were multiprofessional, including neuropsychiatrists, clinical psychologists, psychiatric nurses, educators, psychiatric rehabilitation therapists, and social workers, all trained in early detection and intervention in psychosis. CBT was delivered by clinical psychologists who underwent specific training programmes. Family psychoeducation was provided by mental health professionals—such as psychiatric rehabilitation therapists, psychiatric nurses, educators, and clinical psychologists, who also received specialized training. Each case manager, whether a social worker, psychiatric rehabilitation therapist, psychiatric nurse, or educator, completed specialized training programmes.	Outpatient settings Not mentioned
[19]	NS: 14 sessions for TAU + CBT TD: 6.5–7.5 months DS: One hour per session FS: Fortnightly (±3 days) FU: 12-month follow-up after treatment	TAU: Includes physical care; career counseling; and unstructured information provided to families about disease symptoms, treatment, and prognosis. TAU + CBT: Combines standard treatment with a structured CBT programme divided into two parts: First Part (Sessions 1–9): Focuses on psychoeducation to improve insight into illness, adherence to treatment, early identification of prodromes, relapse prevention, and promoting a healthy lifestyle. Key sessions include: 1. What is the first episode of psychosis? 2. Challenge and importance of insight into vulnerability. 3. Symptom recognition. 4. Prevention of relapses: protective and risk factors. 5. Detection of prodromes. 6. What to do if symptoms emerge again? 7. Treatment adherence. 8. Healthy lifestyles: sleep and sexuality. 9. Healthy lifestyles: substance use. Second Part (Sessions 10–14): CBT for symptom and thought management, including anxiety management techniques and social and problem-solving skills. Sessions include: 10. Anxiety management techniques (I). 11. Anxiety management techniques (II). 12. Social skills: assertiveness techniques. 13. Problem-solving techniques. 14. Final doubts and farewell. Additional Support: Patients can use a telephone helpline between sessions if needed.	Global Assessment of Functioning (GAF) scale Functioning Assessment Short Test (FAST) Clinical Global Impression Scale (CGI) PANSS State-Trait Anxiety Inventory (STAI) Hamilton Rating Scale for Depression (HRSD) Scale Unawareness of Mental Disorder (SUMD) 4-item Morisky Medication Adherence Scale (MMAS)	The treatment is supervised by a clinician trained by a highly experienced expert at the University Hospital of Álava, while evaluations at all centers are conducted by clinicians who are blind to patient allocation. The coordinating group provides therapist training across participating centres via teleconferencing. The training course includes 12 modules and 2 booster sessions, aligning with the 14 sessions of the psychoeducational programme. All participating groups receive the same theoretical content.	Individual and group sessions Outpatient
[85]	NS: 24 sessions + 4 booster sessions of CBT TD: 6-month treatment period DS: 26 h of CBT; 6 h optional family intervention (+ regular communication with family members for those who consented) FS: Weekly individual CBT sessions; monthly family intervention FU: Follow-up visits at 3 months, 6 months, and 12 months	In the initial phase of CBT, patients and therapists collaboratively identify key issues and set goals for the therapy. A personalised maintenance plan is then created. The subsequent phases are dedicated to implementing change strategies as detailed in a published manual, examining historical factors contributing to the onset of first-episode psychosis, and concluding with a phase focused on relapse prevention. The family intervention follows a behavioural family therapy model. It begins with an initial session to conduct an assessment, share formulations, and agree on specific goals and issues. The intervention encompasses psychoeducation, provision of normalising and recovery-oriented information, problem-solving techniques, and strategies for relapse prevention.	PANSS side-effects rating scale First-Episode Social Functioning Scale Questionnaire about the Process of Recovery (QPR) Specific Psychotic Experiences Questionnaire Hospital Anxiety and Depression Scale Alcohol Use Disorder Identification Test Drug Abuse Screening Test NICE-recommended 10-item version of the Autism Spectrum Quotient Economic patient questionnaire EuroQol 5-Dimensions 5-Levels (EQ-5D-5L)	Therapists, who were appropriately trained, received weekly supervision from two MAPS group members (APM and SB). Audio-recorded CBT sessions, conducted with the patient’s consent, were regularly assessed using rotational sampling and rated with the Cognitive Therapy Scale–Revised (by APM and SB) to ensure protocol fidelity.	Outpatient setting Individual
[92]	NS: Not designed to last for a specific number of sessions (determined by client’s preferences, needs, and circumstances). Includes 1 individual session + 10–12 family education sessions + additional sessions as needed. TD: At least two years. DS (Duration of Sessions): Individual resiliency training (IRT)—approximately 1 h per session; family education programme includes various sessions, but exact duration not specified. FS: Weekly or bi-weekly IRT sessions; family education programme includes monthly brief in-person or phone contact, and additional sessions as needed. FU: Not specified.	NAVIGATE is a team-based, multicomponent treatment programme including Family Education Programme, Individual Resiliency Training (IRT), Supported Employment and Education (SEE), Individualised Medication Treatment, and Case Management. Services are customised to client needs, with collaborative goal setting involving clients, team members, and family. Medication Management: Involves monitoring metabolic and cardiovascular risks, adhering to treatment guidelines, coordinating with primary care, and encouraging healthy lifestyles. Family Education Programme: Begins with an initial assessment to understand the client’s perspective, followed by education on psychosis, treatment, and stress management. Includes creating a relapse prevention plan and intensive skills training in communication and problem-solving. IRT: Features goal setting and progress tracking through a modular curriculum. Modules are tailored to client needs, using motivational, psychoeducational, and cognitive–behavioural methods, with home assignments set collaboratively. SEE: Develops work and educational goals based on client preferences, with rapid job or school placement. Provides community-based support, respects client privacy, and includes follow-up to assist with job retention or educational progress.	Not mentioned	Positions are not expected to be full-time, and members may have additional responsibilities. The psychiatrist or nurse practitioner is responsible for medication prescription. Two clinicians with master’s-level degrees manage IRT (Intensive Rehabilitative Therapy) and case management. The specialist in SEE (Specialized Early Education), typically holding a bachelor’s degree, focuses on SEE. The SEE Director, who holds a master’s degree, coordinates and leads the team, supervises IRT clinicians and the SEE specialist, and oversees the FEP programme.	Individual or group Intervention Not mentioned outpatient
[73]	NS: CBT: 20–30 sessions per patient Fip: 10–15 sessions CM: Not specified, but each patient/family has a dedicated case manager TD: 9 months DS (Duration of Sessions): Not mentioned FS: CBT: Weekly sessions for the first 3 months, then fortnightly for the following 6 months Fip: 6 sessions in the first 3 months, then at least 1 session per month during the following 6 months FU: Patients are reassessed after 9 months from the baseline assessment	The multi-component psychosocial intervention comprises: CBTp: Individual therapy focused on managing psychotic symptoms and improving coping strategies. Family Intervention (Fip): Provides education and support to family members, enhancing their understanding of psychosis and improving family dynamics and support systems. Case Management (CM): Offers coordinated care, including assistance with accessing services and ongoing support tailored to individual needs. All components follow NICE guidelines to ensure evidence-based practices in patient and family care.	Test Intelligenza Breve (TIB) Clinical Drug Use Scale (CDUS) PANSS Hamilton Rating Scale for Depression (HAMD) Bech-Rafaelsen Mania Rating Scale (BRMRS) Global Assessment of Functioning (GAF) Psychotic Symptom Rating Scale (PSYRATS) Disability Assessment Scale (DAS) Schedule of Assessment of Insight (SAI-E) Camberwell Assessment of Needs (CAN) World Health Organization Quality of Life (WHO QOL- Bref) Ad hoc schedule for life events Childhood Experience of Care and Abuse Questionnaire (CECA-Q) Parental Bonding Instrument (PBI) Level of Expressed Emotion Scale (LEE) Involvement Evaluation Questionnaire (IEQ-EU) General Health Questionnaire (GHQ) Premorbid Social Adjustment scale; PSA) Life Chart Schedule; LCS) Verona Service Satisfaction Scale, patient version; VSSS-EU) Clinical Assessment in Neuropsychiatry (SCAN)	Professionals received specific training programmes in CBTp (Cognitive–Behavioural Therapy for psychosis), FIp (Family Intervention programmes), and CM (Case Management). Following the training, their competence was assessed, and they were provided with detailed intervention manuals based on international standards. These manuals serve as a guideline for treatment. Professionals are supported by a team of expert psychotherapists assigned to each Community Mental Health Centre (CMHC). Additionally, experimental interventions provided to all patients and their families are supervised by external experts, who hold one-day meetings every two months and are available for regular consultation. TAU is provided by routine public CMHCs.	Urban/rural and mixed zones Outpatient CBT—patient; Family intervention—sessions with each individual family. CM—every patient/family
[68]	NS: 24 sessions TD: 12 weeks DS: 120 min per session FS: Twice per week FU: No follow-up (the follow-up study is underway)	Integration of Cognitive Remediation Approaches The intervention integrates three cognitive remediation approaches: SCIT: Begins with group sessions targeting social cognition domains, including emotion recognition, theory of mind, and interaction skills. The training is structured into three phases: Phase I (Sessions 1–6): Focuses on emotion perception and self-awareness, including emotion mimicry and understanding paranoia. Phase II (Sessions 7–15): Addresses theory of mind, social perception, and attributional biases, teaching participants to differentiate facts from guesses and gather evidence. Phase III (Sessions 16–24): Applies learned skills to real-life situations, emphasizing generalization to daily life. CCT: Uses a strategy-based approach to enhance cognitive functions: Sessions 1–6: Focus on prospective memory, goal setting, and planning. Sessions 7–12: Target conversational and task vigilance, using “self-talk” to maintain focus. Sessions 13–18: Improve verbal learning and memory through information reduction and name-learning. Sessions 19–24: Enhance executive functioning and cognitive flexibility with brainstorming and problem-solving techniques. NEAR: Utilizes individualized iPad training with commercial programmes: Sessions 1–24: Tailor exercises to each participant’s cognitive profile, incorporating games and tasks from BrainHQ, Lumosity, and Games for the Brain. Training is designed to be engaging, with verbal encouragement and metacognitive guidance from therapists. Participants receive a cognitive profile from baseline measures and collaborate with therapists to prioritize cognitive domains for improvement. Each participant is paired with a practice partner to support skill application in everyday life.	Digit Symbol Coding subtest Wechsler Adult Intelligence Scale, 4th edition (WAIS-IV) The Trail Making Test Part A and Part B Wechsler Memory Scale, 3rd edition (WMS-III) Wechsler Abbreviated Scale of Intelligence (WASIis) Delis-Kaplan Executive Function System (D-KEFS) Tower subtest Ambiguous Intentions Hostility Questionnaire-Ambiguous items (AIHQ-A) Facial Emotion Identification Task (FEIT) Beck Cognitive Insight Scale Life Skills Profile-39 (LSP-39) Behaviour Rating Inventory of Executive Function-Adult Version (BRIEF-A) Quality of Life Scale (QLS) Occupational Self-Assessment (OSA) PANSS Depression Anxiety Stress Scale 21-item (DASS-21)	The lead author (OGV) was the primary therapist, trained and supervised by the second and third co-authors (DR and EWT, respectively). Other co-therapists included an occupational therapist, a clinical psychologist, and a staff member from the early intervention centre.	Urban Outpatient Brief, group-based Intervention
[61]	NS: FMSG—16 sessions; Psychoeducation group programme—16 sessions TD: Total intervention—36 weeks DS: FMSG—2 h per session; Psychoeducation group programme—2 h per session FS: FMSG—bi-weekly; Psychoeducation group programme—bi-weekly FU: At one-week, 12-month, 24-month, and 48-month after completing the interventions	Family Support and Psychoeducation Groups FMSG: The FMSG programme follows a structured approach with 16 sessions divided into five stages: Stage 1 (2 Sessions)—Engagement: Introduces the intervention, establishes trust, and defines roles and goals. Discussions include the impact of psychosis and initial family learning. Stage 2 (4 sessions, including 1 with patients)—Awareness: Focuses on mutual psychosocial needs, power dynamics, and family culture. Emphasis on sharing challenges, managing emotions, and understanding psychosis and its effects. Stage 3 (4 sessions, including 2 with patients)—Management: Addresses physical and psychosocial needs, medication adherence, stress management, and effective communication. Includes strategies for home management. Stage 4 (4 sessions)—Caregiving Roles: Enhances coping skills and problem solving through sharing experiences, rehearsals, and practical application of learned skills in real-life situations. Stage 5 (2 sessions)—Termination: Prepares for programme conclusion, reviews achievements, discusses future plans, and explores continued support options. Participants provide input on session topics and modifications. Psychoeducation Group Programme: Consists of 16 sessions with the following themes: Introduction and Goal Setting (2 sessions): Covers basic orientation and goal setting for mental health promotion. Mental Health Skills (5 sessions): Focuses on survival skills, stress management, and mental health promotion. Therapeutic Family Environment (2 sessions): Establishes a supportive family environment. Relapse Prevention and Resilience (5 sessions): Includes problem-solving, interpersonal skills training, and resilience enhancement. Review and Evaluation (2 sessions): Assesses knowledge and skills gained and sets future goals. Both programmes encourage attendance at sessions focused on illness, treatment, medication adherence, and mental health services. Completion is considered for those attending more than seven sessions.	Family Assessment Device (FAD) Family Burden Interview Schedule (FBIS) Family Support Services Index (FSSI) Specific Level of Functioning Scale (SLOF) PANSS	FMSG sessions were co-led by two peer family caregivers with significant caregiving experience. They were trained by researchers through a three-full-day workshop focused on psychoeducation and supportive skills. The peer leaders received additional support from two resource persons (the first author and a rehabilitation nurse specialist) for group resources, development stages, and service referrals. Participants in the psychoeducation group programme received education and psychological support from a psychiatric nurse specialist with five years of experience in mental health education, rehabilitation, and group therapy. This nurse was trained by the research team in a 3-day (20 h) workshop, which included mini-lectures, video presentations, discussions, experience sharing, and supervised practice in group leadership and facilitation. Key topics covered in the programme included harmonious family relationships, caregiving roles and demands, understanding psychosis and its treatments, effective coping and communication skills, and problem-solving and crisis intervention in caregiving.	Outpatient clinic Group intervention
[65]	NS: Multiple Family Groups: Not explicitly stated Individual Therapy (IT): Not explicitly stated TD: IT: 2 years Multiple Family Groups: 18 months DS: Multiple Family Groups: 1.5 h/session IT: Not explicitly stated (primary staff meetings not specified) FS: Multiple Family Groups: Biweekly IT: Biweekly for the first 2 months, then weekly for the following 10 months; weekly support meetings with the primary staff member FU: After 2 years At 2 and 5 years	Intensive Early Intervention vs. Standard Treatment Intensive Early Intervention (IT): This programme spans two years and includes a multimodal approach tailored for first-episode psychosis, integrating assertive community treatment, family involvement, and social skills training. Assertive Community Treatment: An individualized and flexible treatment plan is developed collaboratively with each patient to address their specific needs and enhance adherence. Patients have weekly meetings with their primary staff member, who provides continuous support and coordination, including during hospital admissions and discharges. Family Involvement: Psycho-educational multifamily groups, based on McFarlane’s model, are offered to engage families in problem-solving procedures. Individual family sessions and workshops are also provided to accommodate those unable to attend group sessions. This includes survival skills workshops focusing on illness management and problem-solving. Social Skills Training: This component involves psychoeducation on basic social skills, relapse prevention, medication management, and substance abuse. Social skills training is delivered individually or in groups, depending on the patient’s needs. It emphasizes role-playing, problem-solving strategies, and cognitive therapy principles, targeting skills such as conversation, problem solving, and coping with symptoms. Standard Treatment: Provides basic contact with a community mental health centre without the intensive, multimodal support of the early intervention programme.	Clinical Assessment in Neuropsychiatry (SCAN) Scale for the Assessment of Positive Symptoms (SAPS) Scale for the Assessment of Negative Symptoms (SANS)	The IT team includes a psychiatrist, psychologist, psychiatric nurse, occupational therapist, and social worker. In assertive community treatment, the staff-to-patient ratio is 1:10, with case managers comprising social workers, psychologists, psychiatric nurses, occupational therapists, and a psychiatrist. The OPUS staff comprise a multidisciplinary team that includes a psychiatrist, psychologists, nurses, social workers, a physiotherapist, and a vocational therapist. All team members, except the psychiatrist, serve as primary contacts for patients. The patient-to-staff ratio is 10:1. OPUS staff members are highly educated and experienced in first-episode psychosis, receiving ongoing training and supervision in the core elements of the OPUS treatment to ensure specialized assertive intervention.	Community, outpatient settings. IT—individual for patients and multifamily groups: four to six families Multiple family groups as defined by the McFarlane manual were available. the groups consisted of 4–6 patients with families and 2 therapists simultaneously present
[58]	NS: CBTp and SM: 24 meetings; Group treatments (CBTp and SM): 16–24 h each; Multifamily group: 16 h TD: 3 months DS: 2 h per session FS: Two meetings per week FU: 1-year follow-up	CBTp Programme: This programme integrates psycho-educational methods with cognitive and behavioural techniques. It emphasizes stress management, hypothesis testing, the impact of substance use, and the development of coping skills. Participants are responsible for practicing CBT techniques, engaging in group discussions, and applying strategies to real-life scenarios. SM: This skills training programme focuses on behavioural interventions to teach social skills and manage symptoms to prevent relapse. Responsibilities include role-plays, problem-solving exercises, in vivo practice, and completing homework assignments. AVEC: Utilizing psycho-educational and cognitive–behavioural techniques, this programme addresses stress management, substance use, and coping skills enhancement. Participants are involved in discussions, exercises, and practicing new strategies while collaborating with peers and therapists to achieve both personal and group objectives.	Brief Symptom Inventory (BSI) Multidimensional scale of perceived social support (MSPSS)	Two co-therapists, one from the clinical setting and one from the research team, are involved. Both have experience working with individuals with psychosis but are newly trained in CBTp. They receive intensive 14 h training and fortnightly supervision. All sessions are filmed for supervision and quality control.	Outpatient setting Group (six participants); multi-family group AVEC.
[63]	NS: Not specified; frequency and duration are determined based on individual needs. TD: Adjusted to individual needs; Metacognitive Training: Minimum of 4 months. DS: Psychodynamic Groups: 1 h per session; Metacognitive Training: Not specified; CBT: Not specified; Therapeutic Community Meetings: Not specified; Occupational Therapy: Not specified; Physical Exercise: Not specified; Workshop with the Social Worker: Not specified; Nutritionist Workshops: Not specified FS: Psychodynamic Groups: Three times a week; Metacognitive Training: Once a week; CBT: Once a week; Therapeutic Community Meetings: Twice a week; Occupational Therapy: Three times a week; Physical Exercise: Weekly; Workshop with the Social Worker: Once a week; Nutritionist Workshops: Once every two weeks FU: Not specified	Description: The programme is a comprehensive therapeutic approach incorporating various components designed to address different aspects of mental health and well-being. Components: Psychodynamically Oriented Group Psychotherapy: Focuses on enhancing emotional regulation and reducing symptom severity by exploring intrapsychic experiences and fostering emotional acceptance. Multi-Family Groups: Involves family members to discuss issues such as blame, guilt, negative symptoms, and independence. It aims to provide genuine responses and shared experiences. Cognitive–Behavioural Workshops: Offer psychoeducational sessions on self-concept, emotion recognition, managing negative emotions, relationships, goal setting, stress management, and coping strategies. Metacognitive Training: Engages participants in activities to improve self-awareness and reflective thinking about personal thoughts, feelings, and intentions, enhancing the ability to formulate complex representations. Therapeutic Community Meetings: Facilitates discussions on daily community issues through open dialogue, enhancing communication within the therapeutic setting. Psychoeducation: Provides lectures on illness-related topics with support from the therapeutic team, focusing on acquiring knowledge and presentation skills. Occupational Therapy: Involves therapeutic activities related to self-care, productivity, leisure, arts, sports, education, and social skills, including regular physical exercise and sports games. Socio-Therapy: Offers workshops with a social worker to educate participants about social welfare rights and services. Recreational Therapy: Supports individual development, re-socialization, motivation, and relaxation through a variety of recreational activities. Nutrition Workshops: Educates participants on balanced nutrition, healthy meal preparation, and overall healthy living habits through informative presentations.	Not mentioned	Activities are conducted by a multidisciplinary team consisting of a psychiatrist (group analyst), a nurse (group therapist), two psychologists (cognitive–behavioural therapists, one also a trainee in group analysis), a nutritionist (trainee in group analysis), a social worker, and an occupational therapist (group therapist).	Outpatient Day hospital Individual and group intervention
[69]	NS: 7-session group intervention Engagement phase: 1–2 sessions Psychoeducational: 1–2 sessions Psychosocial management: 3 sessions Psychosocial needs of the caregivers: 2 sessions TD: Not specified DS: 1 to 1.5 h per session FS: Not mentioned FU: assessments at 1 month and 3 months after intervention	Description: The caregiver support programme is structured into four distinct phases aimed at providing comprehensive support to caregivers of individuals with psychosis. Phases: Engagement Phase: Focuses on building mutual support and fostering help-seeking behaviours among caregivers. Key activities include self-introduction, discussion of group norms and goals, and exploring the benefits of the group intervention. Emphasizes creating empathetic relationships, overcoming cultural and linguistic barriers, and promoting group cohesion. Psychoeducational/Psychosocial Management of Illness: Consists of 1–2 sessions dedicated to psychoeducation and peer learning. Covers understanding psychosis, sharing experiences, and improving management strategies. Topics include causes, symptoms, treatment options (pharmacological and psychosocial), managing expressed emotion, and its impact on recovery. Psychosocial Management: Focuses on strategies for handling aggressive and symptomatic behaviours. Includes training on effective communication, problem solving, medication supervision, activity scheduling, and vocational training. Aims to enhance functional outcomes through interventions such as halfway homes, sheltered workshops, and day care centres. Psychosocial Needs of Caregivers: Addresses the health and well-being of caregivers. Topics include stress management, yoga, relaxation techniques, and mindfulness. Encourages strengthening social support networks, seeking help from friends and relatives, and sharing caregiving responsibilities. Termination Phase: Reviews learning experiences and goal achievement. Promotes ongoing support through periodic meetings, telephone calls, or online contact to maintain relationships and continue providing assistance.	Family Questionnaire (FQ) Multidimensional Scale of Perceived Social Support (MSPSS)	Trained psychiatric social worker (first author) with over three years of experience conducting support groups for caregivers of individuals with schizophrenia.	Inpatient Group intervention
[72]	NS: CBT: 12 modules Carer Education Programme: Not specified TD: Not specified DS: CBT: 90 min per session Occupational Therapy: Not mentioned Carer Education Programme: Not mentioned FS: CBT: Weekly Carer Education Programme: Weekends FU: Not specified	Description: The intervention comprises three primary elements: a psychosis education campaign, a rapid assessment service, and specialized recovery-oriented interventions, including CBT, occupational therapy, and carer education. Components: CBT: Modules 1–4: Focus on baseline assessment, outlining programme objectives, and understanding the physiological and behavioural aspects of anxiety, as well as cognitive processes. Modules 5–9: Cover the CBT model of psychosis, introduction to metacognitive training, addressing substance misuse, enhancing social support, and managing social anxiety. Modules 10–12: Address self-esteem and goal setting; medication management; assertiveness, relapse prevention, including early warning signs; and participants’ presentations of their care plans. Occupational Therapy: Aims to assist individuals in achieving meaningful goals related to productivity, social and leisure skills, self-care, and community living. Facilitates reintegration into productive roles, often in collaboration with community agencies. Carer Education Programme: Based on a collaborative approach, incorporating insights from individuals, families, and professionals. Session 1: Understanding psychosis. Session 2: Medical perspectives on psychosis. Session 3: Psychological approaches to treatment. Session 4: Exploring the psychotic experience. Session 5: Addressing issues faced by families. Session 6: Strategies for relapse prevention.	Not mentioned	The team consists of a consultant psychiatrist, project manager, administration officer, clinical nurse specialist, occupational therapist, social worker, psychologist, and clinical fellows.	Community-based, urban region Occupational therapy—individual psychosocial sessions.
[53]	NS: Not specified TD: 7 months therapy window DS: Not specified FS: Approximately fortnightly FU: Face-to-face interviews at baseline, 7, 12, 18, 24, and 30 months; additional telephone calls every 6 weeks for some BPRS items	Description: The intervention integrates cognitive–behavioural family therapy for schizophrenia and family interventions tailored for FEP. It also includes a structured manualized individual therapy approach, informed by previous trials and collaborative therapy models. Components: Individual Therapy (Ryle and Kerr, 2002): Phase 1: Engagement and assessment of recovery status and relapse risk, incorporating elements of cognitive analytic therapy. Phase 2: Collaborative formulation of therapy goals, summarised in a therapeutic letter, influenced by cognitive analytic principles. Phase 3: Focuses on increasing awareness of relapse risks and strategies for mitigation. Phase 4: Identification of early warning signs of relapse and creation of a prevention plan, drawing on Birchwood et al. (1989). Phase 5: Optional modules addressing treatment adherence, substance use, stress management, and co-existing anxiety and depression. Includes review, termination, and scheduling of booster sessions based on EPPIC trials (Edwards et al., 2006; Jackson et al., 2001). Family Therapy: Assessment and Engagement: Review family experiences and dynamics. Evaluation: Examine family communication, stressors, and coping strategies. Psychoeducation: Provide information on relapse risks and early warning signs. Relapse Prevention Plan: Develop a plan to manage relapse risks. Skills Training: Integrate communication skills training and problem-solving strategies as needed, informed by collaborative models (Gilbert et al., 2003).	Montgomery–Åsberg Depression Rating Scale (MADRS) Psychotic Subscale of the (BPRS) Scale for the Assessment of Negative Symptoms (SANS) Social and Occupational Functioning Assessment Scale (SOFAS) Family well-being: General Health Questionnaire (GHQ-28) Experience of Caregiving Inventory (ECI)	The outpatient case managers are fully integrated members of the EPPIC outpatient treatment team. Family therapy is manualized and delivered by a trained family therapist.	Outpatient settings Not mentioned
[31]	NS: Not mentioned TD: Not specified DS: Not specified FS: Individual therapy: 1–2 times a week, weekly, or biweekly Family involvement: Monthly sessions Group therapy: Weekly Cognitive remediation: Weekly FU: 6-month follow-up after discharge from the programme	Description: The POTENTIAL model integrates outreach and engagement with individual, family, and group therapy. It encompasses a range of components designed to provide comprehensive support to patients. Components: Individual Therapy: Utilises an eclectic approach combining psychoeducation, CBT, and psychodynamic and supportive techniques. Tailored to each patient, focusing on acceptance, recovery, and individual strengths and goals. Family Involvement: Includes sessions with a psychiatrist, individual therapist, family therapist, the patient, and parents. Provides monthly support groups for parents. Offers separate sessions with the family and family therapist for psychoeducation, guidance on parenting techniques, effective coping skills, and support in the recovery process. Group Therapy: Facilitates social reintegration through structured sessions with peers who are also recovering from psychotic illnesses. The group model is process-oriented, covering various themes and topics throughout the year. Includes group trips to aid in social reintegration and normalise experiences. Cognitive Remediation: Offered to current and former patients. Led by a clinical psychologist with the assistance of staff, interns, and postdoctoral fellows. Involves computer-based exercises with coaching to enhance performance and implement strategies for improvement.	Not specified	Committee of clinical experts in adolescent and adult psychiatry, along with educators, researchers, and administrators.	Individual and group intervention Outpatient
[83]	NS: Outpatient clinic: Number of sessions not defined Intensive mobile team: Number of sessions not defined Inpatient clinic: Number of sessions not defined TD: 36 months DS: Not mentioned FS: Outpatient clinic: Contact ideally within 48 h; up to two home visits per week in case of crisis; psychoeducation—2 sessions per week Intensive mobile team: Frequency can increase from twice per week to daily monitoring as needed Inpatient clinic: Close interactions with case managers; psychoeducation—2 sessions per week FU: Not mentioned	Psychoeducation Tool: Content: Covers psychosis symptoms, the link between psychosis and cannabis use, medication management, and recovery strategies. Frequency: Conducted twice weekly in both inpatient and outpatient settings. Psychological Intervention for Cannabis Abuse: Focus: Addresses cannabis use disorders in the context of psychosis. Multi-Familial Sessions: Structure: A four-session group addressing essential aspects of psychosis and its treatment. Prospective Monitoring of Medication Side Effects: Objective: Regular assessment and management of medication-related side effects. Cognitive Assessment and Remediation: Purpose: Evaluation and improvement of cognitive functions impacted by psychosis. Supported Employment: Service: Assists patients in obtaining and maintaining employment. Outpatient Clinic: Model: Case management led by nurses or social workers, in close collaboration with psychiatrists. Contact: Initiated within 48 h from hospitals, emergency rooms, general practitioners, or patients’ homes. Caseload: Up to 30 individuals per team. Training: Specialized in assertive case management, following International Early Psychosis Association guidelines. Responsibilities: Coordinate multidisciplinary treatment, conduct home visits during crises (up to twice a week), promote patient engagement, provide psychoeducation, and support relapse prevention and recovery. Operating Hours: Weekdays, with after-hours services through the DP-CHUV emergency facility. Intensive Mobile Team (ACT Team): Function: Engages and assesses treatment-resistant patients, offers intensive temporary treatment including daily medication monitoring, and provides alternatives to hospital admission during relapses. Inpatient Clinic: Approach: Adheres to early intervention principles, employs a low-dose medication strategy. Activities: Includes Tai-chi, creative groups, and interactive psychoeducation sessions. Collaboration: Emphasizes close coordination with case managers and outpatient teams.	Not specified	The team includes case managers, consultant psychiatrists, intern psychiatrists, and psychologists.	Inpatient, outpatient ACT teamIndividual and group sessions
[59]	RAP (Recovery through Activity and Participation) NS (Number of Sessions): ~12 sessions TD (Treatment Duration): Maximum of three months DS (Duration of Sessions): Two hours per session FS (Frequency of Sessions): Twice a week FU (Follow-Up): Not mentioned YES (Youth Education and Support) NS (Number of Sessions): 8 sessions TD (Treatment Duration): Eight weeks DS (Duration of Sessions): Two hours per session FS (Frequency of Sessions): Weekly FU (Follow-Up): Not mentioned Cognitively Oriented Skills Training NS (Number of Sessions): 10 sessions TD (Treatment Duration): Not specified DS (Duration of Sessions): Two hours per session FS (Frequency of Sessions): Weekly FU (Follow-Up): 1 year Family Intervention NS (Number of Sessions): 3 evening psychoeducation workshops, followed by individual support and intervention sessions (number of sessions not mentioned) TD (Treatment Duration): Not specified DS (Duration of Sessions): Not specified FS (Frequency of Sessions): Not specified FU (Follow-Up): 1 year Active Family Support Group NS (Number of Sessions): Not mentioned TD (Treatment Duration): Not mentioned DS (Duration of Sessions): Not mentioned FS (Frequency of Sessions): Not mentioned FU (Follow-Up): 1 year Individual Therapeutic Interventions NS (Number of Sessions): Not mentioned TD (Treatment Duration): Not mentioned DS (Duration of Sessions): Not mentioned FS (Frequency of Sessions): Not mentioned FU (Follow-Up): 1 year Cognitive–Behavioural Interventions NS (Number of Sessions): Not mentioned TD (Treatment Duration): Not mentioned DS (Duration of Sessions): Not mentioned FS (Frequency of Sessions): Not mentioned FU (Follow-Up): 1 year	Assertive Case Management Model: Strategy: Utilizes a modified assertive case management model adapted through psychosocial interventions. Content: Based on the stress-vulnerability model, focusing on comprehensive psychosocial support. Group Intervention—Recovery through Activity and Participation (RAP): Strategy: Provides outpatient-based activities to enhance basic life skills, communication, and mutual support during recovery. Content: Assists both inpatients and outpatients in developing essential skills and managing the transitional phase of recovery from acute psychosis. Family Intervention: Strategy: Includes psychoeducational sessions and individual support for families. Content: Consists of three evening sessions for groups of four to eight families, focusing on education and support. Group Intervention—Youth Education and Support (YES): Strategy: Targets individuals with partially resolved acute symptoms. Content: Sessions cover self-identity, psychosis definition, peer pressure, substance use, family and social relationships, stigma, social skills, and reintegration into school or work. Cognitively Oriented Skills Training: Strategy: Offered to patients post-RAP and YES, focusing on re-entry into education or employment. Content: Includes supportive psychotherapy; problem solving; and cognitive–behavioural interventions for anxiety, depression, and persistent psychotic symptoms. Family Psychoeducation and Support: Strategy: Provides two stages of intervention for younger first-episode patients, including psychoeducation and workshops. Content: Covers diagnostic issues, stigma, substance use, family dynamics, symptom management, and treatment adherence. Features video modules and problem-solving approaches tailored to family needs. Active Family Support Group: Strategy: Facilitates regular meetings for parents and relatives of first-episode patients. Content: Focuses on discussing concerns, providing mutual support, and collaborating with programme staff to plan additional services.	Retrospective Assessment of Onset of Schizophrenia (RAOS) Scale for the Assessment of Negative Symptoms (SANS) Scale for the Assessment of Positive Symptoms (SAPS)	The RAP is conducted twice a week for two hours, lasting up to three months. The YES programme runs for eight weeks, with weekly two-hour sessions involving six to eight patients. Cognitively oriented skills are provided in ten weekly group sessions, each lasting two hours.	Inpatient/outpatient Individual and group sessions
[82]	Visitation and Follow-up NS: Not specified, individual follow-up by psychiatrist, psychologist, social worker, and nurse throughout the programme duration. TD: 5 years DS: Not specified FS: Not specified FU: Clinical, functional, neurocognitive, and genetic assessments at baseline, 6 months, and 1-year follow-up Individual Psychotherapy NS: Not specified TD: 2–3 years DS: 45 min per session FS: Weekly or fortnightly, depending on the characteristics of each patient FU: Not specified Group Psychotherapy NS: Not specified TD (Treatment Duration): 2–3 years DS: 1 h per session FS: Weekly FU: Not specified	Individualized Treatment Planning: Strategy: Tailors treatment to individual needs, incorporating therapeutic requirements for both patients and caregivers. Content: Based on Alanen et al.’s principles, interventions are flexible and case-specific, focusing on understanding patient and family experiences through a psychotherapeutic lens. Assertive Community Treatment (ACT): Strategy: Provides intensive, community-based support and follow-up through home visits. Content: Aims to strengthen the therapeutic alliance and engage patients struggling with mental health services. Therapeutic Modalities: Strategy: Includes both individual and group psychotherapy, involving relatives when appropriate. Content: Individual Psychotherapy: Enhances therapeutic alliance, insight, and emotional management; integrates psychotic symptoms; and recognises prodromal signs. Group Psychotherapy: Fosters communication, insight, treatment adherence, emotional expression, and daily relationship management. Psychotherapeutic Treatments for Families: Unifamily Group: Focuses on resolving communication conflicts, identifying relapse triggers, and improving treatment adherence. Multifamily Group: Enhances family communication, identifies early relapse signs, and facilitates experience sharing among families. Family Psychoeducation: Builds trust, provides information about psychosis, highlights its impact on family dynamics, identifies relapse signs, and addresses emotional challenges. Coordination and Quality Assurance: Strategy: Ensures effective care through staff coordination and continuous quality assessment. Content: Adapts therapeutic modalities to meet patient and family needs, ensuring comprehensive support and adaptive care.	Not specified	Alanen et al. provided training directly to the clinicians involved in the programme. This training, informed by the pioneering work of Yung et al. and based on recommendations from a clinical guide for early psychosis by the Spanish and Catalonian governments, ensured consistency with formative experiences.	Outpatient Family and patient
[91]	Computerized CR NS: Not specified TD: 12 weeks DS: Not specified FS: Not specified FU: Follow-up at 12 weeks CBTp NS: Not specified TD: 42 weeks DS: Not specified FS: Not specified FU: Follow-up at 42 weeks	CR: Strategy: Utilises the “Computerized Interactive Remediation of Cognition-Interactive Training for Schizophrenia” (CIRCUITS) software. Content: The programme involves tasks designed to enhance skills such as sustained attention, working memory, and planning. Tasks progress in difficulty and complexity, with modifications made to fit individual needs, aiming to maintain motivation and engagement. CBTp: Strategy: Delivered by therapists specifically trained in CBTp, under the supervision of senior CBTp practitioners. Content: Adheres to NICE guidelines for CBTp, focusing on evidence-based practices to address psychosis.	Psychotic Symptoms Rating Scales (PSYRATS) PANSS Calgary Depression Scale for Schizophrenia (CDSS) Rosenberg Self-Esteem Scale (RSES) Social and Occupational Functioning Assessment Scale (SOFAS) Wechsler Adult Intelligence Scale (WAIS) Wechsler test of adult reading (WTAR) Wisconsin Card Sort Task (WCST)	A time-matched social worker with 1 week of specific training to deliver Circuit.	Homebased CR is provided in patients’ homes or service bases

## Appendix E

**Table A5:** Programme names and intervention objectives (categorized by single/multicomponent).

Intervention Type/Programme Name Uni/Multicomponent	Intervention Objective
**Uni component**	
[50] PSBI—Problem-Solving Bibliotherapy Intervention	Improve the caregiving experience and reduce psychological distress among caregivers in the PSBI group; achieve lower levels of expressed emotion and enhance overall health in the PSBI group.
[55] CBT-based intervention self-directed problem-solving bibliotherapy	Promote carers’ well-being and support them in their caregiving roles.
[81] PIENSA—programa de Intervención en Psicosis Adolescente; psychoeducational problem-solving	Structured group meetings can reduce anxiety, encourage emotional expression and processing, foster collaborative problem solving, enhance a sense of control over challenging situations, and develop realistic action plans for managing specific problems.
[86] CRT	Enhance cognitive function and support functional recovery in neuropsychiatric disorders.
[87] CRT—individual	Enhance cognitive deficits by teaching information processing strategies via structured mental exercises.
[78] NEAR—CRT; EPIP—assertive community treatment (case management); psychoeducation and individual and group psychotherapy (only CRT under study)	Improve patients’ cognitive abilities.
[60] CCT	Develop new cognitive habits that generalize to cognitive performance and meaningful real-world outcomes (compensatory cognitive training approaches teach cognitive strategies as ways of working around cognitive impairments).
[93] CR	Improve cognitive functioning in the CR group.
[89] MOL—method of levels therapy transdiagnostic cognitive therapy	Address goal conflict and facilitate resolution through an innate trial-and-error system called “reorganization” by maintaining awareness of the conflict’s source, helping to reorganize goals, and enabling individuals to regain control.
[80] SocialMIND	Cultivate an acceptance-based, non-judgemental approach towards both one’s own experiences and those within interpersonal relationships.
[62] Psychoeducation	Improve carers’ knowledge about psychosis.
[52] MFG—multiple family group education programme—psychoeducation	Improve families’ perceptions of their knowledge and understanding regarding mental illness and its treatment (practical/economic skills, intellectual/theoretical components, and personal/interpersonal growth).
[90] Cognitively focused brief group intervention—psychoeducation	Enhance illness models by reducing perceived negative consequences and blame. Improve overall understanding of the illness, including its timeline and treatments.
[88] CR Intervention—cognitive therapy-based	Reduce trauma symptoms and depression while improving self-esteem.
[54] LifeSPAN therapy—cognitive-oriented therapy (EPPIC)	Enhance clinical and administrative mechanisms for better detection and monitoring of high-suicide-risk patients.
[51] ACE—active cognitive therapy for early psychosis—CBT	Treat patients in the acute phase of FEP using CBT to achieve faster reductions in both positive and negative symptoms and to improve functioning more rapidly compared to a befriending group; reduce hospitalisations and shorten the length of hospital stays relative to the befriending approach.
[67] CBT	Provide initial evidence on the effectiveness of adapted CBT for reducing positive symptoms; achieve remission of negative symptoms and depression; and enhance overall psychosocial functioning and quality of life.
[77] CBT	Decrease depressive symptoms, boost self-esteem, alleviate positive psychotic symptoms, and enhance overall functioning.
[71] CBT	Symptom improvement following group CBT.
[79] CBT for cannabis cessation	Achieve higher rates of cannabis cessation and improved clinical and functional outcomes compared to the control group post-treatment.
[57] CBT for social anxiety	Reduce social anxiety sustained at 3- and 6-month follow-ups; decrease positive and negative symptoms, with enhanced recovery and functioning.
[76] ACT-DL—acceptance and commitment therapy in daily life—CBT	Modify psychotic experiences, social functioning, and general psychopathology with evidence of sustainable change and its underlying mechanisms in daily life.
[84] Actissist—digital health intervention (DHI)—computerised	Track distressing experiences and deliver real-time management strategies that enhance both the speed and quality of recovery in psychosis, surpassing the outcomes of conventional treatments.
**Multicomponent**	
[56] CAT—cognitive adaptation training, ABCR—action-based cognitive remediation—computerised	CAT—Address cognitive difficulties and motivational issues using home-based environmental supports and conducting weekly home visits; ABCR—Implement computerised cognitive training and real-world practice exercises.
[70] CReSt-R—cognitive remediation and social recovery in early psychosis—combines CRT (computerised interactive remediation of cognition training for schizophrenia)—CIRCuiTS—with social recovery therapy (SRT)	Maximise the cognitive and functional gains from psychological interventions, focusing on social and occupational functioning as well as social cognition.
[64] NEUROCOM—CR + OPUS treatment (social skills training, patient psychoeducation and psychoeducational family treatment)	NEUROCOM: enhance cognitive function, psychiatric symptoms, and overall functional capacity; OPUS treatment: alleviate psychotic symptoms and assist in coping with the illness.
[49] COPE—recovery-focused therapy; cognitive–behavioural + psychoeducation + case management	Facilitate the individual’s adjustment and prevent or alleviate secondary morbidity following the FEP.
[74] Pr-EP—comprehensive intervention package: pharmacological treatment, psychosocial interventions (CBT: psychoeducational sessions for family members, recovery-oriented CM)	Stabilise symptoms through medication; enhance coping strategies via CBT; educate families to improve support networks. Promote long-term recovery with case management; foster personal and social recovery.
[66] CM + Psychoeducation + CBT	Prevent relapse; diminish the risk of transition to chronic diseases; decrease functional impairment.
[75] Re-Arms—reggio emilia at-risk mental states: CBT+ CM + psychoeducation	Prevent the progression of disease; improve symptom management; increase understanding through psychoeducation; reduce DUP; promote personal and social recovery; decrease stigma; and enhance social inclusion.
[19] CBT + psychoeducation	Enhance functioning, treatment adherence, and awareness of the illness; achieve a greater reduction in depressive, negative, and general psychotic symptoms following treatment.
[85] CBT + psychoeducation	Clinical improvement of the patient (psychoeducation, provision of normalising information and recovery-oriented information, problem solving, and relapse prevention planning).
[92] NAVIGATE—family education programme + individual resiliency training (IRT) + supported employment and education (SEE) + case management	Deliver a comprehensive intervention tailored to the specific treatment needs of families and individuals recovering from an FEP—family education programme—for families. The objectives include establishing collaborative relationships between the family and treatment team, instilling hope for recovery, educating about psychosis and its treatment, enhancing communication, reducing family stress, boosting support for the client’s goals and treatment participation, and preventing relapses. IRT—For individuals, the intervention aims to help clients achieve personal recovery goals, educate them about psychosis and its treatment, process their experience of the psychotic episode, improve illness self-management (including relapse prevention and coping strategies), reduce substance abuse, enhance social support and quality of relationships, increase resilience and well-being, and improve overall health. SEE—The intervention seeks to support clients in obtaining and maintaining competitive employment and enrolling in mainstream education programmes; individualised medication treatment aims to reduce symptoms while minimising side effects and adverse health outcomes.
[73] CBT + CM + Fip—family intervention for psychosis	Enhance functioning, adherence to treatment, and understanding of the condition; achieve a more substantial reduction in depressive, negative, and general psychotic symptoms following treatment.
[68] NEAR—cognitive remediation + compensatory cognitive training (CCT) + social cognition and interaction training (SCIT)	SCIT aims to address a comprehensive range of social–cognitive aspects; CT focuses on specific cognitive areas—such as prospective memory, attention, learning/memory, and executive functioning—with the goal of helping patients develop practical cognitive strategies and establish meaningful, long-term habits; NEAR seeks to build upon cognitive improvements.
[61] FMSG—family psycho-education group + support group	FMSG aims to enhance family functioning and reduce re-hospitalisation rates among caregivers and patients; it seeks to alleviate family burden and address patients’ psychotic symptoms and overall functioning while also improving the utilisation of mental health services.
[65] OPUS—integrated treatment (assertive community treatment + social skills training + multifamily groups)	Improve negative, psychotic, and disorganized symptoms
[58] CBTp + SM + AVEC (psychoeducation + cognitive/behavioural techniques)	Group treatments CBTp and SM aim to enhance multiple protective factors: skills, social competencies, family and social support, adaptive strategies, self-esteem, stress management, and medication compliance; AVEC aims to empower families to support each other and provide information on various aspects of psychosis.
[63] Comprehensive therapeutic programme (day hospital)—psychodynamically oriented group psychotherapy + multi-family groups + cognitive–behavioural workshops + metacognitive training + psychoeducation + occupational therapy + socio-therapy + recreational therapy + workshops	Provide a comprehensive therapeutic approach using all effective methods to achieve and sustain remission, recovery, insight, and treatment adherence.
[69] Psychoeducational/psychosocial management of the illness	Improve social support and reduction in EE.
[72] DETECT—CBT + occupational therapy + carer education programme	DETECT aims to reduce delays in receiving effective care and provide tailored treatment for the early phase of psychosis; CBTp helps individuals understand their experiences and reduce stress, minimising the impact of symptoms on cognitive and social functioning; occupational therapy helps individuals regain their occupational identity; the carer education programme provides an understanding of the condition and available treatments.
[53] EPPIC—multi-modal therapeutic intervention relapse prevention treatment (RPT) + individual and family-based CBT	Interventions designed to prevent relapse following FEP.
[31] POTENTIAL (multidisciplinary clinical programmes)—individual therapy (psychoeducation + CBT + psychodynamic and supportive techniques + psychoeducation for family) + group therapy (social reintegration + CR)	POTENTIAL programme aims to prevent or lessen the development of chronic mental illness in young adults; collaborate with the patient, family, and support network to create a personalised recovery plan that motivates the patient to engage in treatment and effort; facilitate the patient’s reintegration into work, school, and social activities to prevent relapse; and provide ongoing therapeutic and educational support for both the patient and their family, including a monthly support group for parents.
[83] TIPP-Lausanne—assertive community treatment + CM + psychoeducation	Enhance continuity of care between inpatient and outpatient settings by establishing a specialised team for early psychosis treatment with dedicated outpatient and inpatient units; reduce the DUP and lower inpatient admission rates; provide tailored family support through multi-family groups; implement ongoing clinical monitoring; and develop an integrated research programme.
[59] PEPP—recovery through activity and participation (RAP) + YES + cognitively oriented skills training + family intervention support group + individual therapeutic interventions + cognitive–behavioural interventions	RAP intervention focuses on assessing and strengthening basic life skills, communication, and mutual support during the recovery phase from acute psychosis.; YES programme pairs relevant psychoeducational themes; patients who complete RAP and YES and are preparing to re-enter school or work receive cognitively oriented skills training. Family interventions, adapted from Anderson and colleagues’ model, provide early-phase illness information for younger first-episode patients. An active support group for families, including parents and relatives, offers emotional support and collaborates with programme staff on additional services. Individual therapy includes supportive psychotherapy and problem-solving to address daily challenges and psychosis-related trauma, while cognitive–behavioural interventions target anxiety, depression, and persistent symptoms. All group and family interventions follow established manuals.
[82] Integrated need-adapted treatment	Individual psychotherapy aims to build a strong therapeutic alliance, enhance insight, and recognise early warning signs and high-stress situations to prevent future relapse; it also focuses on understanding and integrating psychotic symptoms with the patient’s experience, encouraging emotional expression, and improving emotional management; group psychotherapy seeks to enhance communication with peers, improve insight and treatment adherence, and foster a sense of connection through shared experiences. It helps patients find meaning in their psychotic experiences, express their emotions and feelings about social and family relationships, and address group dynamics to better manage daily interactions.
[91] CR + CBT	Improve participants’ cognitive skills through CR, thereby facilitating various CBTp processes beyond symptom reduction, such as developing a shared understanding of the patient’s problems (an agreed formulation); additionally, the intervention aims to reduce delusions and hallucinations more effectively and earlier when CBTp is preceded by CR, and to enable CBTp to be completed more quickly or achieve greater progress before completion by enhancing cognitive skills with CR.

## Appendix F

**Table A6:** Grouped table of scales and articles.

Primary Focus	Scale	Articles
Experience and Family Functioning	- Experience of Caregiving Inventory (ECI) - Family Assessment Device (FAD) - Family Burden Interview Schedule (FBIS) - Family Questionnaire (FQ) - Family Support Services Index (FSSI) - Family Well-being: General Health Questionnaire (GHQ-28) - Kessler Psychological Distress Scale (K10) - Level of Expressed Emotion Scale (LEE) - Multidimensional Scale of Perceived Social Support (MSPSS) - Parental Bonding Instrument (PBI) - Perceived Criticism Scale - Short Form Health Survey (SF-12)	[50,53,58,61,62,69,73,84]
Quality of Life	- Clinical Outcomes in Routine Evaluation—Outcome Measure (CORE-OM) - EuroQol 5-Dimensions 5-Levels (EQ-5D-5L) - General Health Questionnaire (GHQ) - Modular System for Quality of Life (MSQoL-R) - Outcome Rating Scale (ORS) - Psychological Outcome Profiles (PSYCHLOPS) - Quality of Life Scale (QLS) - Questionnaire about the Process of Recovery (QPR) - Recovery Assessment Scale (RAS) - Session Rating Scale (SRS) - World Health Organization Quality of Life, Brief Version (WHO QOL-Bref)	[49,53,54,57,66,67,68,71,73,85,86,87,89,91]
Cognitive and Intelligence Assessment	- 5-Item Version of the Hinting Task - Behavior Rating Inventory of Executive Function—Adult Version (BRIEF-A) - Beck Cognitive Insight Scale - Birchwood Insight Scale - Brief Assessment of Cognition in Schizophrenia (BACS) - California Verbal Learning Test - Cambridge Neuropsychological Test Automated Battery (CANTAB) - Cognitive and Intelligence Assessment (CogState) - Delis–Kaplan Executive Function System (D-KEFS) Tower Subtest - Danish Adult Reading Test (DART; i.e., Danish version of NART) - Digit Span Forwards and Back Test - Digit Vigilance Test (DVT) - Explanatory Model Scale (EM) - Eyes Test - Facial Emotion Identification Task (FEIT) - Hopkins Verbal Learning Test—Revised (HVLT–R) - Integration/Sealing Over (I/SO) Measure - MATRICS Consensus Cognitive Battery (MCCB) - Measurement and Treatment Research to Improve Cognition in Schizophrenia Consensus Cognitive Battery (MCCB) - Modified Six-Elements Test - Montreal Cognitive Assessment (MoCA) - Need for Cognition Scale (NCS) - Penn Emotion Recognition Test (ER-40) - Premorbid Assessment Scale (PAS) - Retrospective Assessment of Onset of Schizophrenia (RAOS) - Rey Auditory Verbal Learning Test - Rey Osterreith Complex Figure (ROCF) - Scale to Assess Unawareness of Mental Disorder (SUMD) - Schedule of Assessment of Insight (SAI-E) - Stroop Color and Word Test (STROOP) - The Trail Making Test Part A and Part B - The Wide Range Achievement Test (WRAT III; Wilkinson) - Test Intelligenza Breve (TIB) - Timeline Follow Back - Trail Making Test: Part B - Digit Symbol Coding Subtest Wechsler Adult Intelligence Scale, 4th ed. (WAIS-IV) - Wechsler Abbreviated Scale of Intelligence (WASI) - Wechsler Adult Intelligence Scale (WAIS) - Wechsler Adult Intelligence Scale-III (WAIS-III-UK) - Wechsler Memory Scale (WMS) - Wechsler Test of Adult Reading (WTAR) - Wisconsin Card Sort Test (WCST)	[49,56,57,59,60,64,66,67,68,70,71,73,78,80,84,86,87,91,93]
Psychiatric Symptoms	- Affective Disorders and Schizophrenia for School-Age - Alcohol Use Disorder Identification Test (AUDIT) - Ambiguous Intentions Hostility Questionnaire (AIHQ) - Bech–Rafaelsen Mania Rating Scale (BRMRS) - Beck Anxiety Inventory (BAI) - Beck Depression Inventory (BDI) - Beck Depression Inventory (BDI) - Beck Hopelessness Scale (BHS) - Brief Negative Symptom Scale (BNSS) - Brief Psychiatric Rating Scale (BPRS) - Brief Psychiatric Rating Scale (BPRS) (Expanded Version 4) - Brief Symptom Inventory (BSI) - Children—Present and Lifetime Version (K-SADS-PL) - Clinical Global Impression for Bipolar Disorder (CGI-BD) - Clinical Assessment in Neuropsychiatry (SCAN) - Clinical Drug Use Scale (CDUS) - Comprehensive Assessment of At-Risk Mental States (CAARMS) - Drug Abuse Screening Test (DAST) - Drug Use Disorders Identification Test (DUDIT) - General Symptom Index (GSI) of the SCL-90-R - Knowledge About Psychosis Scale - Positive and Negative Syndrome Scale (PANSS) - Premorbid Social Adjustment Scale (PSA) - Hamilton Rating Scale for Depression (HRSD) - Hamilton Depression Rating Scale (HDRS-21) - Hospital Anxiety and Depression Scale - Negative Symptom Assessment (NSA) - Psychotic Subscale of the BPRS - Psychotic Symptoms Rating Scales (PSYRATS) - Reasons for Living Inventory (RFL-24) - Royal Park Multidiagnostic Instrument for Psychosis (RPMIP) - Scale for the Assessment of Negative Symptoms (SANS) - Scale for the Assessment of Positive Symptoms (SAPS) - Self-Evaluation of Negative Symptoms (SENS) - Specific Psychotic Experiences Questionnaire - Suicide Ideation Questionnaire (SIQ) - Suicide Intent Scale - Young Mania Rating Scale (YMRS)	[19,49,50,51,53,54,56,58,59,60,61,62,64,66,67,68,70,71,73,75,76,77,78,79,80,81,84,85,90,91,93]
Social and Occupational Functioning	- Bell Lysaker Emotion Recognition Task (BLERT) - Camberwell Assessment of Needs (CAN) - Children’s Global Assessment Scale (C-GAS) - Disability Assessment Scale (DAS) - Emotion Recognition Task (ERT) - Empowerment Rating Scale (ERS) - First Episode Social Functioning Scale - Functioning Assessment Short Test (FAST) - Global Assessment of Functioning (GAF) - Goal Attainment Scale (GAS) - Hinting Task - Involvement Evaluation Questionnaire (IEQ-EU) - Life Skills Profile-39 (LSP-39) - Occupational Self-Assessment (OSA) - Multnomah Community Ability Scale (MCAS) - Personal and Social Performance (PSP) - Reading the Mind in the Eyes Task - Reorganisation of Conflict Scale (ROC) - Social and Occupational Functioning Assessment Scale (SOFAS) - Social Behaviour Schedule—Total Problem Score on SBS - Social Functioning Scale (SFS) - Social Problem-Solving Inventory-Revised Short Form (SPSI-R) - Specific Level of Functioning Scale (SLOF) - UCLA Social Attainment Survey (SAS) - University of California, Performance Based Skill Assessment—Brief Version (UPSA-B)	[19,49,51,53,54,55,56,57,60,61,64,66,67,68,70,71,73,75,76,77,78,80,81,84,85,86,87,88,89,90,91,93]
Anxiety and Depression	- 13-item Beck Depression Inventory (BDI) - Beck Anxiety Inventory (BAI) - Beck Depression Inventory (BDI-II) - Beck Hopelessness Scale (BHS) - Brief Social Phobia Scale (BSPS) - Calgary Depression Scale for Schizophrenia (CDSS) - Calgary Depression Scale (CDS) - Depression Anxiety Stress Scale 21-item (DASS-21) - General Health Questionnaire (GHQ)-12 - Hamilton Anxiety Scale (HAM-A) - Hamilton Rating Scale for Depression (HAMD) - Hospital Anxiety and Depression Scale (HADS) - Montgomery–Åsberg Depression Rating Scale (MADRS) - Mindful Attention and Awareness Scale (MAAS) - Social Phobia Inventory (SPIN) - State-Trait Anxiety Inventory (STAI) - The Impact of Events Scale - The Social Interaction Anxiety Scale (SIAS) - Worry Subscale of the Somatic, Cognitive and Behaviour Anxiety Inventory	[19,49,53,54,57,60,62,64,65,66,67,68,71,73,76,77,80,84,85,88,91,93]
Self-Esteem and Self-Perception	- Ad Hoc Schedule for Life Events - Internalized Stigma of Mental Illness (ISMI) - Intrinsic Motivation Inventory for Schizophrenia Research - Life Events Questionnaire - Rosenberg Self-Esteem Scale (RSES) - Self-Esteem Rating Scale—Short - Self Esteem Scale (SES) - Self-Report Problem Solving Rating Scale (SRPSRS) - The Robson Self Esteem Questionnaire (SCQ)	[53,54,57,62,64,65,66,70,73,77,86,87,88,91]
Insight and Awareness of Illness	- Adapted Illness Belief Questionnaires - Birchwood Insight Scale - Chinese Ways of Coping Questionnaire (CWCQ) - Integration/Sealing Over (I/SO) Measure - Level of Expressed Emotion (LEE) - Scale to Assess Unawareness of Mental Disorder (SUMD)	[19,49,58,59,62,66,71,72,73,79,85,90]
Family Functioning Assessment	- Family Burden Interview Schedule (FBIS) - Family Questionnaire (FQ) - Family Support Services Index (FSSI) - Parental Bonding Instrument (PBI)	[53,61,62,69,73]
Services & Resources	- 4-item Morisky Medication Adherence Scale (MMAS) - Cannabis Use Problems Identification Test (CUPIT) - Client Service Receipt Inventory (CSRI) - Economic Patient Questionnaire - European Addiction Severity Index (EUROP-ASI) - Verona Service Satisfaction Scale, Patient Version (VSSS-EU)	[4,5,16,26,27,34]
Other	- Brief Adherence Rating Scale—Clinical Global Impression Scale (CGI) - Childhood Experience of Care and Abuse Questionnaire (CECA-Q) - Clinical Global Impression Scale (CGI) - Cognitive Therapy Rating Scale (CTRS) - Experience Sampling Method (ESM) - Experience Sampling Method (ESM) on a Smartphone-based App (the PsyMate™ app) - Functioning Assessment Short Test (FAST) - Life Chart Schedule (LCS) - Medication Adherence Rating Scale (MARS) - Time Use Survey - Timeline Follow Back (TLFB)	[4,5,9,10,13,14,16,20,25,26,27,28,33,40]
